# Regional distribution and losses of end-of-life steel throughout multiple product life cycles—Insights from the global multiregional MaTrace model

**DOI:** 10.1016/j.resconrec.2016.09.029

**Published:** 2017-01

**Authors:** Stefan Pauliuk, Yasushi Kondo, Shinichiro Nakamura, Kenichi Nakajima

**Affiliations:** a Faculty of Environment and Natural Resources, University of Freiburg, Freiburg D-79106, Germany; b Graduate School of Economics, Waseda University, Tokyo, Japan; c Center for Material Cycles and Waste Management Research, National Institute for Environmental Studies, Tsukuba, Japan

**Keywords:** Circular economy, MaTrace, Steel recycling, Closed-loop recycling, Circularity metric

## Abstract

Substantial amounts of post-consumer scrap are exported to other regions or lost during recovery and remelting, and both export and losses pose a constraint to desires for having regionally closed material cycles. To quantify the challenges and trade-offs associated with closed-loop metal recycling, we looked at the material cycles from the perspective of a single material unit and trace a unit of material through several product life cycles. Focusing on steel, we used current process parameters, loss rates, and trade patterns of the steel cycle to study how steel that was originally contained in high quality applications such as machinery or vehicles with stringent purity requirements gets subsequently distributed across different regions and product groups such as building and construction with less stringent purity requirements. We applied MaTrace Global, a supply-driven multiregional model of steel flows coupled to a dynamic stock model of steel use. We found that, depending on region and product group, up to 95% of the steel consumed today will leave the use phase of that region until 2100, and that up to 50% can get lost in obsolete stocks, landfills, or slag piles until 2100. The high losses resulting from business-as-usual scrap recovery and recycling can be reduced, both by diverting postconsumer scrap into long-lived applications such as buildings and by improving the recovery rates in the waste management and remelting industries. Because the lifetimes of high-quality (cold-rolled) steel applications are shorter and remelting occurs more often than for buildings and infrastructure, we found and quantified a tradeoff between low losses and high-quality applications in the steel cycle. Furthermore, we found that with current trade patterns, reduced overall losses will lead to higher fractions of secondary steel being exported to other regions. Current loss rates, product lifetimes, and trade patterns impede the closure of the steel cycle.

## Highlights

• Up to 50% of steel consumed today lost in obsolete stocks or landfills by 2100.
• Trade-off between low losses and high-quality applications of steel was quantified.
• Reduced steel losses during recycling lead to higher secondary steel exports.
• Current loss rates, lifetimes, and trade patterns impede closure of metal cycles.
• Results obtained with MaTrace Global, a Markov-chain model of steel consumption.

## Graphical abstract

**Figure:**
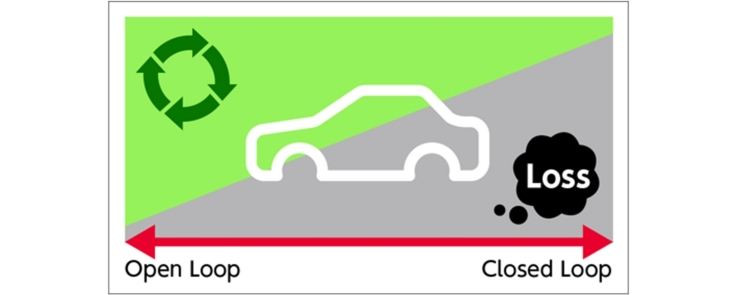

## 1 Introduction

### 1.1 The determinants of material recycling

Recycling is a key strategy to reduce dependency on mineral resources, GHG emissions in primary material production, and other negative impacts in the mining and material industries (Damgaard et al., 2009, Ignatenko et al., 2008, Johnson et al., 2008). Recycling can lead to closed material cycles, where little or no primary material production is needed, and it is therefore a central aspect of paradigms for a sustainable economy, including industrial ecology (Ehrenfeld, 2002, Jelinski et al., 1992) and the circular economy (European Commission, 2015, Ghisellini et al., 2015, Yuan et al., 2006). The extent of metal recycling is constrained by the amount of available end-of-life (EoL) products, from which postconsumer scrap can be recovered. Three factors therefore determine the contribution that recycling can make to closed material cycles: the growth rate of in-use stocks, which determines to a large degree how much material has to be produced from virgin resources (Gordon et al., 2006, Müller et al., 2006); the ability of the waste management industries to recover scrap of sufficiently low contamination (Reuter et al., 2006a); and the ability of the remelters to produce secondary materials of high quality and with low loss rates (Reuter et al., 2006b, van Schaik et al., 2002).

### 1.2 System models to study material recycling

**Fig. 1:**
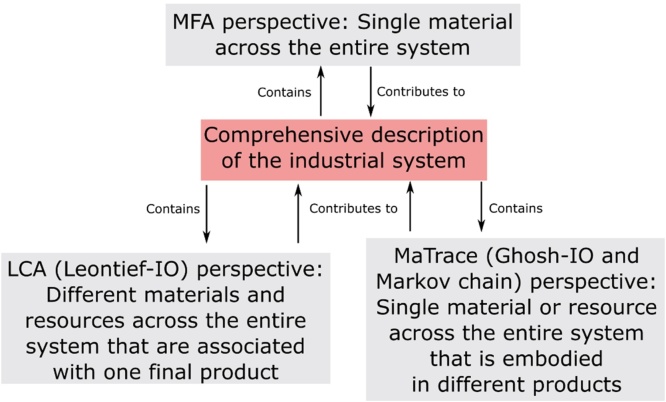
Overview of the different perspectives on the industrial system offered by MFA, LCA (Leontief IO), and MaTrace.

To study the impact of stock growth and technological change in the waste management and remelting industries on material flows, resource use, emissions, and environmental product footprints in a systematic manner, a detailed and balanced description of the industrial metabolism is necessary (Majeau-Bettez et al., 2014). Such a description would have to respect multiple balances for the different materials and chemical elements so as to serve as a sound empirical basis for the computation of the different process and product indicators (Schmidt et al., 2012). Such a detailed and balanced description of the industrial metabolism does not exist yet, but several of its physical and monetary aspects are accounted for and assessed by the methods material flow analysis (MFA), life cycle assessment (LCA), and Markov chain modelling. Each of these methods offers a certain perspective on the industrial system and uses specialized databases. Thus the methods contribute to the comprehensive description of the industrial metabolism (Fig. 1), and allow for the study of recycling in particular.

Dynamic material flow analysis (MFA) is used to model the impact of stock growth and the deployment of different recycling technologies on the material cycles. Within MFA, stock growth is studied by prospective dynamic stock models that relate population growth and lifestyle to levels of in-use stocks and that apply stock-driven modelling (Müller, 2006) to build scenarios for future metal cycles, including the levels of primary and secondary production (Liu et al., 2012, Oda et al., 2013, Pauliuk et al., 2013, Pauliuk et al., 2012). The impact of scrap recovery and remelting technologies on future material cycles is studied by prospective dynamic material cycle models as well (Davis et al., 2007, Hatayama et al., 2010, Liu et al., 2012, Milford et al., 2013, van Ruijven et al., 2016, Wang et al., 2015). The existing models have in common that they describe national, regional, or global material cycles at their respective scale for the different model time steps; they can quantify recycling levels, loss rates, and the levels of application of recycled material in different product categories for the entire system. They do not, however, trace back the origin of secondary material to its source products and can therefore not be applied to research questions pertaining to the distribution of scrap from different products onto different applications. In addition, MFA does not allow to trace the different primary and secondary materials embodied in final consumer products. To quantify embodied recycled materials and to understand how a certain unit of recycled material passes through different products, regions, and stages of the material cycle a different perspective on the industrial metabolism is necessary.

Embodied materials and the generation of recycled materials from end-of-life products are studied by taking a life cycle perspective on products and services. This is the research domain of life cycle assessment (LCA), and calculations of embodied materials and emissions are based on a Leontief input output model (Heijungs and Suh, 2002). A third systems perspective on recycling is offered by tracing a specific unit of material that participates in the life cycles of different products. This supply-driven perspective is taken by the Ghosh-input-output (IO) model (Guerra and Sancho, 2011), absorbing Markov chains (Duchin and Levine, 2013, Eckelman and Daigo, 2008, Eckelman et al., 2012, Yamada et al., 2006), and MaTrace (Nakamura et al., 2014). The supply-driven perspective is complementary to the life cycle perspective. The full-scale, life cycle, and material unit perspectives are complementary and they allow researchers to look at material cycles from different angles (Fig. 1).

### 1.3 The closure of the steel cycle and the research gap

Steel is the most widely used metal by far and due to the importance of steel for industry, trade, resource use, and emissions to the environment the steel cycle and steel-containing products have been studied extensively from an MFA perspective, e.g., (Cullen et al., 2012, Müller et al., 2006, Wang et al., 2007) and an LCA perspective, e.g., (Kim and Wallington, 2013, Worldsteel Association, 2011, Worldsteel Association, 2010). The MaTrace perspective (Nakamura et al., 2014), has to this day only been applied to Japan, and there is currently no model that allows to physically trace a certain unit of a recycled material through global supply chains. (There are applications of monetary IO tables to the problem, e.g., Moran et al. (2014)). Moreover, a quantitative indicator for the contribution of a certain unit of material to the closure of a material cycle is lacking.

### 1.4 Goal and scope of this work

MaTrace allows for tracing a certain unit of a recycled material through the supply chain (Nakamura et al., 2014). The model combines a dynamic stock model of the use phase of metal with a linear model of the waste management industries, the remelting processes, the manufacturing sectors, and the markets for EoL products, scrap, secondary metals, and final products. The modelling principle of MaTrace is closely related to absorbing Markov chains (Yamada et al., 2006) and the Ghosh IO model (Duchin and Levine, 2013, Duchin and Levine, 2010). The original version of MaTrace was applied to Japan and thus it disregards the distribution of steel scrap onto different regions via trade. A detailed scenario analysis with MaTrace to investigate the impact of different technological options on keeping steel service at high levels throughout the 21st century is lacking. To fill this gap we formulated the following research questions:

• How does the product and regional distribution of a unit of steel consumption change over time under different improvement options in steel recycling?
• How big is the loss of steel in the recycling loop under different recycling technology deployment scenarios?
• How can the contribution of a certain unit of material to the closure of a material cycle be quantified and what can do we learn from this indicator?

To answer these questions, we present and apply the methodology of MaTrace Global, a multiregional extension of MaTrace with global scope. We then present scenario results for the regional and product distribution of steel in passenger cars and machinery consumed in 2015 in different countries, including the US, Japan, China, and Germany, until 2100. We propose a performance indicator for the circularity of material use throughout several life cycles, *Circ(2100)*, that can be calculated from the scenario results of MaTrace Global. We analyze how current and anticipated technological options can change the product distribution of the steel in the future and reduce losses to landfills and slag piles und thus improve circularity. Finally, we discuss the technological and political implications of our work.

## 2 Methodology

### 2.1 MaTrace Global

We extended the regional scope of the MaTrace model (Nakamura et al., 2014) to cover the whole world economy in 25 regions. The model equations, the assumptions for the parameters, and the data sources used are presented in detail in the Supplementary information (SI1). The complete model (Python script with the model calculations and Excel file with the data and scenario parameters) is available as Supplementary material (SI2). Here we describe the main characteristics of MaTrace Global and propose a metric for assessing the circularity of material use from the perspective of a unit of metal passing through different applications throughout its life cycle.

**Fig. 2:**
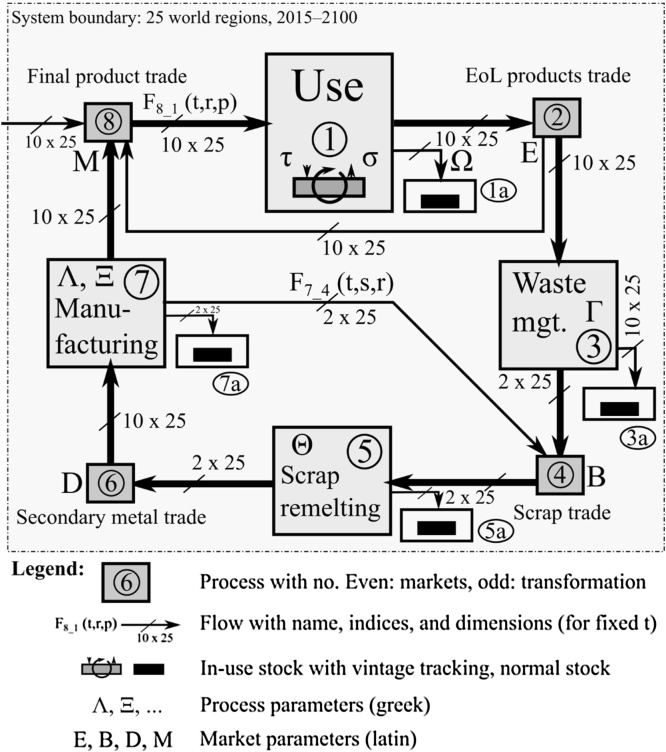
System definition and dimensions of system variables of MaTrace Global.

MaTrace Global contains eight processes, four of which are transformation processes (1, 3, 5, and 7, including the use phase), and four of which are markets (2, 4, 6, and 8) (Fig. 2). The model considers 86 model years *t* and age-cohorts *t’* (2015 ≤ t ≤ 2100, 2015 ≤ t’ ≤ 2100, t’ ≤ t), distinguishes between 10 product groups (*p*), 25 regions (*r*), two types of scrap (*s*, fabrication and postconsumer scrap), and two types of secondary steel making (*m,* electric arc furnace (EAF) and basic oxygen furnace (BOF)). In the case of scrap going into the BOF-steel route we only traced the recycled fraction in the BOF steel and disregarded the primary component.

We compiled available data on trade of three sample EoL products (EoL passenger car de-registered in the US, Japan, and Germany), as well as trade of scrap, secondary metal, and final products, to derive multiregional distribution matrices (market shares or sales coefficients) whose coefficients tell the percentage of an incoming EoL product/scrap/metal/final product in region *r* that goes to the waste management industries/remelters/manufactures/use phase in region *r*’. EoL product trade data for the passenger vehicles *E* were directly obtained from national statistical offices and trade of scrap metal *B* was extracted from BACI, a refined version of the UN Comtrade database (Gaulier and Zignago, 2010). For the multiregional sector split *D* and the product distribution matrix *M* data from the EXIOBASEv2 multiregional supply and use table (Wood et al., 2014) were used. As with the single-region version of MaTrace (Nakamura et al., 2014) *D* was calculated from the material content of products *C* and the final demand vector, and *C* was determined using EXIOBASE data and the WIO-MFA approach (Nakamura et al., 2007). Process data including product lifetimes, the loss rates during the four transformation stages, the recovery rates of postconsumer scrap in the waste management industries, and the recovery rates of fabrication scrap in the manufacturing industries were compiled from previous work (Murakami et al., 2010, Oguchi et al., 2010, Pauliuk et al., 2013) and aggregated or disaggregated to fit the regional classification of MaTrace Global.

A baseline scenario that extrapolates current process parameters and trade patterns until the end of the century was constructed, albeit some anticipated improvements in the recovery of postconsumer scrap were included (WorldSteel Association, 2008). We do not consider the baseline case as particularly likely or unlikely; it merely shall serve as reference case. In the baseline we included the observation that postconsumer scrap, whose largest contributors are steel scrap flows from vehicles and machinery (Allwood et al., 2012, Ohno et al., 2014) is remelted in the EAF route and subsequently used in construction only (Cullen et al., 2012), which is a consequence of the relatively high concentration of tramp elements, especially copper and tin, in the scrap flows and the higher tolerance levels for these elements in construction steel compared to steel for vehicle and machinery applications (Hatayama et al., 2014, Savov et al., 2003).

Starting from the baseline, we constructed a set of improvement scenarios, some of which are listed in Table 1 and all of which are described in SI1. Improvement options include the previously identified material efficiency strategies such as lifetime extension (Milford et al., 2013), fabrication yield improvements (Milford et al., 2011), and the assumption that due to better sorting and disassembly routines most postconsumer scrap can be recycled in the BOF route. We also analyzed the impact of changing the coefficients that allocate steel to different products by using coefficients that distinguish between applications for primary and secondary steel. These coefficients were obtained by disaggregating steel flows in EXIOBASE into BOF and EAF steel, using detailed Japanese data on the share of the EAF and BOF routes in the steel consumption of the different sectors (Ohno et al., 2015) as a proxy for all world regions. Table 1 lists the different improvement options used for the sensitivity analysis (results shown in SI1) and for a set of combined scenarios, which are analyzed below.

### 2.2 The circularity indicator of metal use

In the circular economy, the “value of products, materials and resources is maintained in the economy for as long as possible, and the generation of waste [is] minimised” (European Commission, 2015). While the circular economy concept is gaining traction in the business world and among policy makers, there is no established metric to characterize the circularity of the economic system or a subset thereof. Each of the three perspectives shown in Fig. 1, the MFA, LCA, and MaTrace perspectives, can contribute circularity indicators that provide specific insights into the structure of material cycles. In MFA a number of well-defined recycling metrics exist (Graedel et al., 2011), and these are static material cycle indicators that in most cases do not distinguish between different material qualities. The recently proposed Material Circularity Indicator (Ellen MacArthur Foundation, 2015) also assumes a static perspective, but from a product life cycle point-of-view, and it can therefore be seen as LCA-type indicator. To add the MaTrace perspective and to overcome the limitations of the hitherto proposed circularity metrics, we propose a material-based circularity indicator that refers to a certain amount of material originally consumed in a product at a time *t_0_*.

To assess the circularity of metal use one needs to know the purity, quality, and recoverability of the metal in the use phase, which is the source of future scrap flows. We therefore propose to simply use the mass of the material in the system, broken down into the shares *x_U_* of different product applications and sinks, and weighted by a factor *w* that measures purity, quality, and recoverability (0 ≤ w ≤ 1). The share of the material that is present in form of functional and highly recoverable applications is assigned *w* *=* *1*, whereas losses in landfills or slag piles are weighted at *w* *=* *0* (Eq. (1)).

(1) $$w=\left\{ \begin{array}{l} 1,\text{ use phase}\\ 0,\text{ losses} \end{array}\right.$$

We can then define the circularity index *Circ(T)* as a relative measure of the cumulative mass of steel present in the system over a certain time interval in terms of an ideal reference case, where all steel remains in functional applications throughout the entire accounting period (Eq. (2), cf. also SI1).

(2) $$Circ(T)=\frac{1}{\left(T-t_{0}\right)}\cdot \int_{t_{0}}^{T}\langle x_{U}^{}(t),w\rangle dt$$

The bracket operator denotes the scalar product of *x_U_* and *w*, as both are vectors whose elements are indexed by the different application and loss categories of steel. *Circ(T)* assumes its maximal value 1 if no losses occur over the entire calculation period. Similar to the global warming potential, Circ(T) depends on the time horizon *T*, and the indicator adds a temporal perspective to complement the material cycle performance indicators based on annual snapshots (Graedel et al., 2011, Jiu-ju et al., 2008). In this paper we calculate *Circ(2100)* for each scenario (Table 1 and Fig. 5).

## 3 Results

**Fig. 3:**
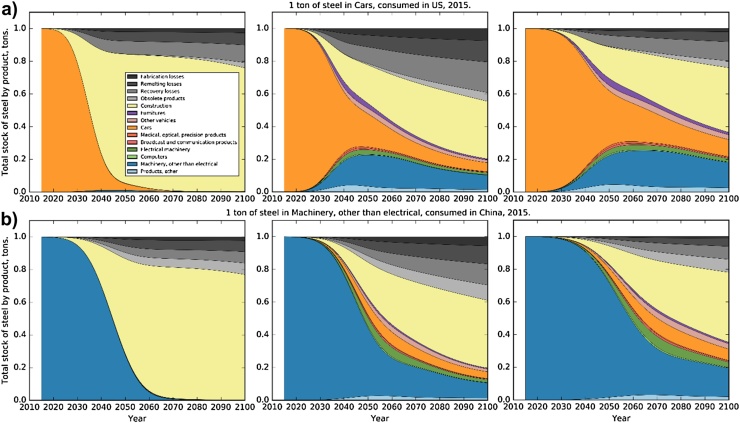
Tracing of 1 ton of steel consumed in 2015 until 2100. Breakdown of the ton into 10 product categories and 4 loss categories. (a) Steel in passenger vehicles consumed in the US in 2015. (b) Steel in machinery consumed in China in 2015. **Left:** Baseline scenario, **middle:** ClosedLoop scenario, **right:** ClosedLoop_Lt_HighRecovery scenario.

Under business-as-usual (BAU) assumptions most of the high quality steel (cold-rolled steel) initially produced for automotive or machinery applications will be recycled into concrete reinforcement bars or other hot-rolled construction steel throughout large parts of the 21st century, while up to about 20% will get lost during the recovery and remelting stages (Fig. 3, left side). This finding is consistent for all regions, and two examples are shown in Fig. 3. More steel can be kept in higher-quality applications (Fig. 3, middle), provided that the waste management industries will be able to extract scrap of sufficiently high quality from EoL products. Losses will more than double, though, because the typical lifetime of high quality applications is only between 30% and 50% of the typical building lifetime, and thus, the steel needs to be remelted more often. Fig. 3 illustrates the trade-off between low losses, which favors application of secondary steel in construction, and the desire to keep steel in high-quality applications.

Material efficiency strategies, such as lifetime extension and higher scrap recovery rates, can alleviate the situation. In the best case we found, which includes a reduction of remelting yield losses by 50%, 20–22% of the steel will get lost by 2100, 38–42% will be used in construction, and 35–40% will remain in high-quality applications (Fig. 3, right side).

**Fig. 4:**
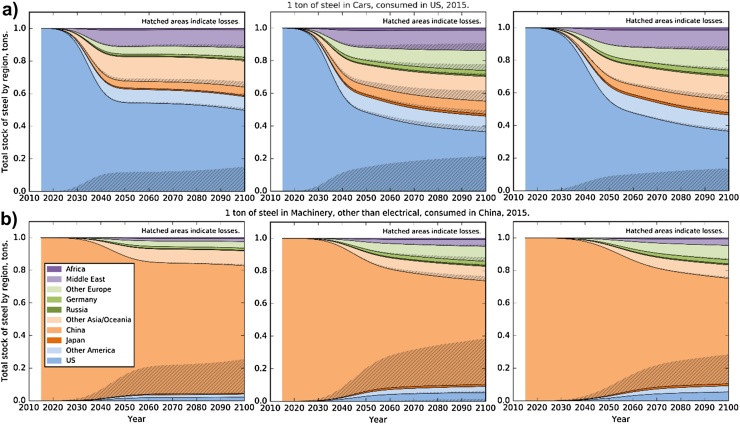
Tracing of 1 ton of steel consumed in 2015 until 2100. Breakdown of the ton of steel into 10 aggregated regions, where hatched areas indicate losses that accumulate within the different regions. (a) Steel in passenger vehicles consumed in the US in 2015. (b) Steel in machinery consumed in China in 2015. **Left:** Baseline scenario, **middle:** ClosedLoop scenario, **right:** ClosedLoop_Lt_HighRecovery scenario.

The future regional distribution of the presently consumed steel depends on the region of consumption and its trade patterns at the different stages of the recycling loop (Fig. 4). With current trade patterns, for the US only 40–50% of the steel in registered passenger cars will still be in the country by 2100, and the fraction of the metal that still provides useful service can be lower than 20% (Fig. 4a middle). Most of the losses will accumulate in the US, and the products that contain the remelted steel are distributed across the world regions, where Africa, Other Europe, and China receive roughly equal shares of about 15%. In relative terms the Chinese recycling loop is less connected to the rest of the world that the US steel cycle, and therefore, between 75 and 85% of the steel consumed in a machine in China in 2015 will still be in the country by 2100 under the assumption of constant trade patterns. Most of the losses, between 20 and 30% by 2100, will also accumulate in China. SI1 contains analogous graphs for a passenger car consumed in Japan and Germany that show similar results.

**Fig. 5:**
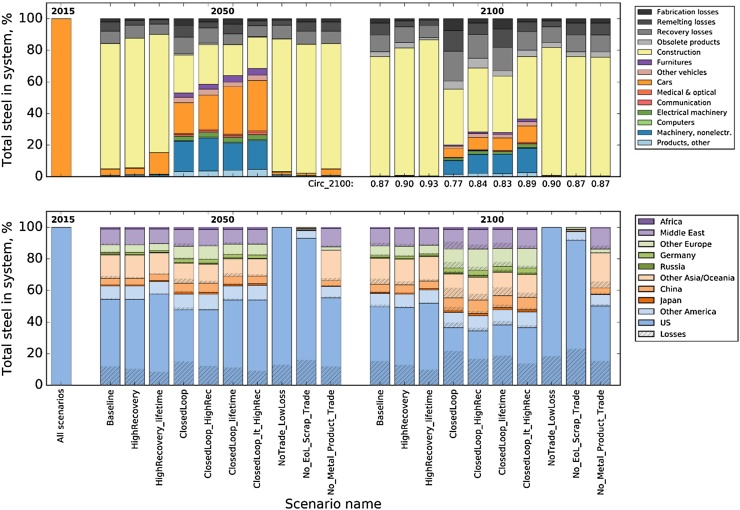
Tracing 1 ton of steel, consumed as passenger vehicle in the US in 2015, until 2100. Product category (**top**) and regional breakdown (**bottom**), for 2015 (Baseline), and 2050 and 2100 for the combination scenarios described in Table 1.

For the combination scenarios presented in Table 1, Fig. 5 contains two snapshots from the time series area plots, one for 2050 and one for 2100 that show the product and regional composition of the steel stock. Similar plots for the results of the sensitivity analysis of individual parameter variations are displayed in SI1.

Changing only the recovery rates or the product lifetimes leads to smaller losses and a shift from buildings to cars of not more than 10 percentage points in 2050 (scenarios HighRecovery and HighRecovery_lifetime). The low sensitivity of the baseline scenario to improvement in the recovery rates has two reasons: First, the improvements were assumed to happen gradually until 2050, which means that by the time the vehicle will be scrapped, around 2030, the improvement potential, which is modest anyway, will have been seized by less than 50%. The second reason is that once the steel ends up in construction there is not much additional turnover before 2100. Only if there is a combined change in the recycling route and the allocation of secondary steel to new products, one can expect a substantial increase of the share of the steel remaining in high-quality applications (scenarios whose names start with ‘ClosedLoop’). The NoTrade_LowLoss scenario shows a possible situation where the steel stays within the US, and closed-loop recycling remains at a high level. Trade in EoL products and scrap are the two main routes of steel leaving the US (scenario No_EoL_Srap_Trade), while product and secondary metal trade play a minor role (No_Metal_Product_Trade).

The baseline scenario has a *Circ(2100)* indicator of 87%, which means that the cumulative residence time of the ton of steel in the use phase from 2015 to 2100 amounts to 87% of the theoretical maximum cumulative use time. The sensitivity analysis does not lead to large changes in the *Circ(2100)* metric, with two exceptions: For Sens_Scrap_BOF and Sens_SectorSplit the indicator decreases to about 0.8 because both cases entail an increased use of secondary steel in high quality applications, which have relatively short lifetimes and thus lead to higher cumulative losses. Assigning specific weighting factors to the different steel qualities during calculation of the Circ(2100) metric would allow analysts to better take into account the benefits of high quality steel (cf. page 26 of SI1). Only the scenarios that focus on subsequent construction application and that increase recovery rates or reduce scrap show a *Circ(2100)* of 0.9 or more. The scenarios where secondary steel is diverted into high quality applications score lower on the *Circ(2100)*-scale because of the higher losses due to shorter lifetimes. The indicator values thus reflect the trade-off between the two strategies of trying to keep as much steel in the use phase as possible and trying to keep steel in high quality applications. Another important finding is that due to the trade structure of the US, secondary steel that gets diverted away from construction applications into automobiles and machinery has a higher change of being exported. This means that the in the ClosedLoop scenarios, not only will the losses be higher but also the fraction of the steel remaining in the US will be lower compared to the scenarios where secondary steel is applied mostly in construction. Similar findings were made for Japan and Germany (cf. SI1, Figs. S8 and S9). These results hint at an additional trade-off between strategies for keeping steel in high-quality applications and strategies for keeping steel within the region.

## 4 Discussion

The scenario results show that with current product lifetimes, recycling technology, and trade patterns the cumulative loss for a unit of steel during the 21st century can be as high as 50% and up to 95% of the steel can leave the region of original consumption. Losses are significantly lower if steel is ‘down-cycled’ to construction steel. If the steel is to stay in high-quality applications, the loss fraction can be reduced to 20–25% by higher scrap recovery rates, longer product lifetimes, lower scrap remelting yields, and increased use of the steel scrap in the BOF route. Loss shares below 20% can only be achieved if steel scrap from EoL vehicles is used in buildings and infrastructure with prolonged lifetime. The trade-offs between strategies aiming at low losses, strategies for keeping steel within the region, and strategies for keeping steel in high-quality applications put a severe limit to closed metal cycles. We discuss the technological and political implications of this finding and give an outlook on future research on the circularity of material use.

### 4.1 Uncertainty and reliability of the results of MaTrace Global

The scenarios generated by MaTrace global build on assumptions and scenario values for the future development of the different model parameters. The model results exhibit both, parameter uncertainty and scenario uncertainty. Unlike parameter uncertainty, which describes the variance of the true parameter values around the reported mean value, scenario uncertainty implies that the mean values themselves are subject to significant change over time. Due to its magnitude, the scenario uncertainty of the model parameters has the largest impact on the scenario results, and we applied a sensitivity analysis to quantify its effect on the results.

Because scenario uncertainty, for which a probability distribution cannot be established, dominates the variance of the model results, one cannot assess the likelihood of the different scenarios produced by MaTrace Global.

Second to the scenario uncertainty, the parameter uncertainty needs to be kept in mind when interpreting the results. While there is reasonably accurate data for the industrial processes and trade patterns related to scrap, steel, and final products, only few data and estimates are available for the formation of stocks and the trade of obsolete or end-of-life products. In Germany, for example, in some years the fate of up to 50% of EoL cars was not reported (Kohlmeyer, 2012), and trade patterns of EoL vehicles change rapidly from one year to the next one due to changing political circumstances and changes in export regulations. More complete and reliable EoL product statistics will help to produce more robust (rather than just plausible) scenarios for MaTrace and other prospective material flow models. The assumption of constant technology and trade patterns is a major limitation that MaTrace shares with other bottom-up approaches, especially attributional LCA. An option to overcome this limitation is discussed in Section 4.4.

### 4.2 Technical aspects of closing material cycles

Resource shortage is not a pressing issue for steel, but every gram of steel lost needs to be replaced by primary production, as global stocks keep growing (Milford et al., 2013). It is therefore worthwhile to investigate a wide spectrum of options to increase the circularity of steel use. Moreover, steel has an established recycling system and secondary steel can accept higher levels of impurities than for example aluminium (Gaustad et al., 2012, Løvik et al., 2014). The steel cycle can therefore serve as a test case for a closed material cycle. Losses of metal through dissipation, sorting, and remelting are unavoidable consequences of the second law of thermodynamics (Reuter et al., 2006b). Still, there is some potential for further reducing these losses through better value chain and in-use stock management. WorldSteel, for example, expects EoL recovery rates of steel to increase from present levels and this assumption is part of our baseline scenario (WorldSteel Association, 2008). Additional improvements may be possible, especially if shredding and subsequent sorting is displaced by dismantling and design for disassembly (Ferrão and Amaral, 2006), or replaced by advanced sensor-based sorting systems (Gaustad et al., 2012). Economic incentives such as scrap premiums can help to reduce the formation of obsolete product stocks and increase fabrication yield loss recovery. Detailed empirical research on the effectiveness and optimal design of such incentives is necessary. For some metals, including aluminium, the potential for applications of lower material quality is limited (Modaresi and Müller, 2012) and it is worth investigating under which circumstances future climate and resource policy should discourage low-quality applications of secondary metals. The development of remelting processes with lower loss rates or the fine-tuning of existing ones is a central contribution the steel industry can make to close the steel cycle. Material efficiency strategies that avoid remelting, including re-use and remanufacturing, are an alternative (Allwood et al., 2012, Milford et al., 2013). These strategies are thus part of the spectrum of strategies to close metal cycles. Multi-material recycling systems, which allow for separating and recovering large pure fractions of not only one but many of the materials contained in modern consumer products, would be the next logical step.

### 4.3 MaTrace Global, the *Circ(2100)* metric, and resource policy

Due to the long lifetime of steel-containing products the steel cycle changes slowly and it may take long before resource policies will show a measureable effect. To facilitate policy development and deployment it is therefore important to identify useful steel cycle performance indicators that can bridge the gap between the need of policy makers for setting short- and mid-term targets (2020–2030) and the long-term response of the steel cycle. The loss rates in the use phase, the waste management industries, steel remelting, and manufacturing may represent such indicators.

Moreover, resource policy makers should be aware of the trade-offs between low losses and high quality applications and between regional dispersion and high quality applications in the steel cycle, which were identified during the scenario analysis. The existence of these trade-offs suggests that there is no ‘silver bullet’ for the transition to closed metal cycles. Instead, a wider spectrum of strategies needs to be explored, including the six material efficiency strategies identified earlier (Allwood et al., 2012, Carruth et al., 2011, Milford et al., 2013), material-efficient remelting technologies, and incentives to reduce formation of obsolete stocks and losses during scrap recovery.

In developing countries the infrastructure for EoL treatment is known to be less material-efficient than in developed ones (Li et al., 2013, Secretariat of the Basel Convention, 2011). It could be possible to prolong the useful life of a material by improving the EoL infrastructure of countries that are the final destination of EoL product trade. From a policy point of view, a quantification of the impact of improved EoL treatment infrastructure and an identification of the final destinations itself could be of importance.

Dynamic material cycle models like MaTrace Global can be applied to both questions as they can assess the impact of the different development paths for loss rates and material efficiency. From their results indicators on the circularity of steel use, including the *Circ(2100)* metric, can be derived. In this paper only the most basic version of *Circ(2100)* was applied. In a next step, one could distinguish between material use in high-quality applications including vehicles, machines, and appliances, denoted as case (a), applications of lower quality, here construction steel, case (b), and losses at the different stages of the system (c). One would then set the high-quality applications as reference and define *w[a]* *=* *1*, set *w[c]* *=* *0*, and set *w[b]* *=* *x*, where *x* is the relative weight of the down-cycling application in terms of the original applications (a). The choice of *x* would be subjective to a large extent but could be defined by industry best practice based on research and sensitivity analysis. The choice of subjective weighting factors is also common in life cycle assessment, especially for the determination of endpoint indicators in life cycle impact assessment. Contrarily, weighting is not necessary in most material flow analysis-related research. As soon as aggregate performance indicators for materials or material cycles are to be derived, however, weighting may become necessary also in MFA. A good example for weighting in MFA is the criticality metric developed by Graedel et al., 2015, Graedel et al., 2012, who present a basic version of their criticality indicator in their publications and mention that industrial users of the methodology are free to choose their own weighting factors between the different components of the metric.

For a more differentiated determination of the *Circ(2100)* indicator the weighting factors could be derived in an objective manner directly from the physical properties of the material in its different applications. Possible properties include: the main metal content (e.g., for copper), the yield strength, (for steel), the tramp metal content (for steel and aluminium), or the ductility (for aluminium). Eligible properties need to be evaluated by experts for the recycling of the different metals.

### 4.4 MaTrace Global and other prospective assessment models

MaTrace Global is a supply-driven model that distributes the material in a given quantity of EoL products onto refinement processes, products, and regions. It complements demand-driven assessments based on the Leontief-IO approach, including LCA, that estimate the total industrial activity required to produce a unit of consumption. While LCA allows modelers to study different material inputs for a single product, MaTrace studies how a single material is distributed across different products. MaTrace thus helps modelers to depict the complexity of the recycling network. MaTrace faces similar limitations as LCA regarding the indeterminacy of future technological and trade pattern developments and regarding scalability. Both models deal with these challenges in different ways. While attributional LCA considers recent process data to determine a timeless life-cycle inventory, even if a product’s life cycle extends far into the future, MaTrace explicitly considers time and deals with the indeterminacy through scenario and sensitivity analysis. As LCA is increasingly being linked to macroeconomic scenarios generated, for example, by integrated assessment models (IAM) (Daly et al., 2015, Hertwich et al., 2015, Wiebe, 2016), MaTrace Global could also benefit from using trade patterns and technology choices from such scenarios as part of its database. This link between MaTrace and IAM scenarios could help to overcome a central limitation of MaTrace Global, the fixed technology assumption. The current setup of MaTrace Global allows for applying the model to small amounts of metal only, as a supply-driven model implicitly assumes that there is a sufficiently high demand to ‘absorb’ the different target products. On the large scale this is often not the case, and especially the sector split parameter D should then be modified according to future demand scenarios. This way, the features of supply- and demand-driven models could be combined to study linkages in socioeconomic metabolism in a more consistent manner which would make future material cycle scenarios more robust.

To facilitate evaluation and interpretation MaTrace results for a given year can be visualized using material stock demographics (Cabrera Serrenho and Allwood, 2016) to show the vintage structure of a unit of material in a future year.

### 4.5 MaTrace Global for multiple products and materials

Metal cycles are coupled at several places: the mining stage (Graedel et al., 2015, Tisserant and Pauliuk, 2016), the alloying stage (Johnson et al., 2008, Ohno et al., 2016, Reck et al., 2010), the product stage, and the waste management stage (Allwood et al., 2011, Reck and Graedel, 2012, Reijnders, 2016). For the steel cycle the study of several metals in parallel is important to understand the recycling and losses of steel alloying elements (Nakajima et al., 2013, Reck et al., 2010), the accumulation of copper and tin in recycled steel (Nakamura et al., 2012, Savov et al., 2003), co-use of metals (Daigo et al., 2014), and the substitution between different materials (Kim and Wallington, 2013, Modaresi et al., 2014). Studies of coupled material cycles are an upcoming topic in MFA (Løvik et al., 2015, Løvik et al., 2014, Nakajima et al., 2013, Ohno et al., 2014), and future versions of MaTrace Global should cover different metals and their alloying elements to allow for studying the interrelations between different recycling systems. Such studies should apply the findings of criticality assessment, especially regarding the substitutability of materials (Nuss et al., 2014), and in turn, they could inform dynamic criticality assessments or criticality scenarios. Challenges that can be better understood with multi-material modelling include changing embodied carbon emissions associated with material substitution and their impact on emissions reduction targets for specific materials, the co-production of metal ores in mines, and the optimal recovery of materials and alloys from the waste streams.

## Figures and Tables

**Table 1:** Scenario definition and the *Circ(2100)* indicator for a passenger vehicle in the US consumed in 2015. The first block of scenarios lists examples of the sensitivity analysis and the second block describes the scenarios analyzed below in Fig. 3, Fig. 4, Fig. 5. The scenarios analyzed in Fig. 3, Fig. 4 are highlighted. The complete list of scenarios and the results of the sensitivity analysis are presented in SI1.

*Values apply for a passenger vehicle, consumed in the US in 2015.

## Appendix A Supplementary data

We provide Supplementary material that contains the model equations, the documentation of data sources and treatment, and additional results (SI1). We also provide the complete MaTrace Global model, including the data and configuration file (Excel) and the model scripts (Python) (SI2).
