# Supplementary material for: Regional distribution and losses of end-of-life steel throughout multiple product life cycles—Insights from the global multiregional MaTrace model
Source: Resour Conserv Recycl. 2017 Jan;116:84–93. doi: 10.1016/j.resconrec.2016.09.029 (PMC5302007; doi:10.1016/j.resconrec.2016.09.029)
Supplement: Supplementary file 1 [file mmc1.pdf]

Resources Conservation and Recycling

Regional distribution and losses of end-of-life steel throughout multiple product life cycles — Insights from the global multiregional MaTrace model

Stefan Pauliuk,[1,] Yasushi Kondo,[2] Shinichiro Nakamura,[2] and Kenichi Nakajima[3]*

[1] Faculty of Environment and Natural Resources, University of Freiburg, Germany
[2] Graduate School of Economics, Waseda University, Tokyo, Japan
[3] Center for Material Cycles and Waste Management Research, National Institute for Environmental Studies, Tsukuba, Japan

Supplementary Material 1 (of 2)

To whom correspondence should be addressed:

*) Stefan Pauliuk
Faculty of Environment and Natural Resources, University of Freiburg, D-79106 Freiburg, Germany
stefan.pauliuk@indecol.uni-freiburg.de; phone+49-761-203-98726; fax +49-761-203-3600

CONTENTS

CONTENTS 2
METHODOLOGY 3
The system definition of MaTrace Global 3
The indices, system variables, and model parameters of MaTrace Global 4
The model equations of MaTrace Global 7
DATA SOURCES AND MODEL PARAMETERS 9
Mean lifetime, τ(r,p) 9
Standard deviation of mean lifetime, σ(r,p) 10
Obsolete stock formation rate, Ω(t,r,p) 11
Trade of EoL products, E(t,r,r',p) 12
Recovery rate of scrap from EoL products, Γ(t,r,p,s) 13
Allocation of scrap to refinement processes and regions, B(t,s,r,m,r') 14
Remelting yield, Θ(t,m,r) 17
Allocation of secondary metal to products and other regions, D(t,m,r,p,r') 17
Fabrication yield, Λ(t,r,p) 22
Fabrication scrap recovery rate, Ξ(t,r,p,s) 23
Trade of final products, M(t,r,r',p) 23
THE CIRCULARITY PERFORMANCE METRIC Circ(t): 24
SCENARIO DEFINITION 27
Sensitivity analysis 27
Scenarios for improving steel cycle circularity. 28
ADDITIONAL RESULTS 29
GUIDE TO THE MATRACE GLOBAL MODEL IN PYTHON 39
REFERENCES 40

METHODOLOGY

We present the system definition of MaTrace Global, define the indices and system variables, and introduce the model equations and model parameters.

The system definition of MaTrace Global

Figure S1 shows the system definition of MaTrace Global. The world is divided into 25 regions (Table S2), and we consider 10 product groups (Table S3), two types of steel scrap (Table S4), and two remelting routes (Table S5). The use phase (1) contains a dynamic stock model that tracks the different age-cohorts through their lifetimes. The other processes respond instantaneously and the model equations that involve parameters for the processes (2)-(8) are solved year-by-year. The four transformation processes (1), (3), (5), and (7) generate losses, which are called obsolete stocks (losses in the use phase), landfilled postconsumer metal waste (losses during scrap recovery from end-of-life (EoL) products), slag piles (losses during remelting), and fabrication yield losses (during manufacturing).

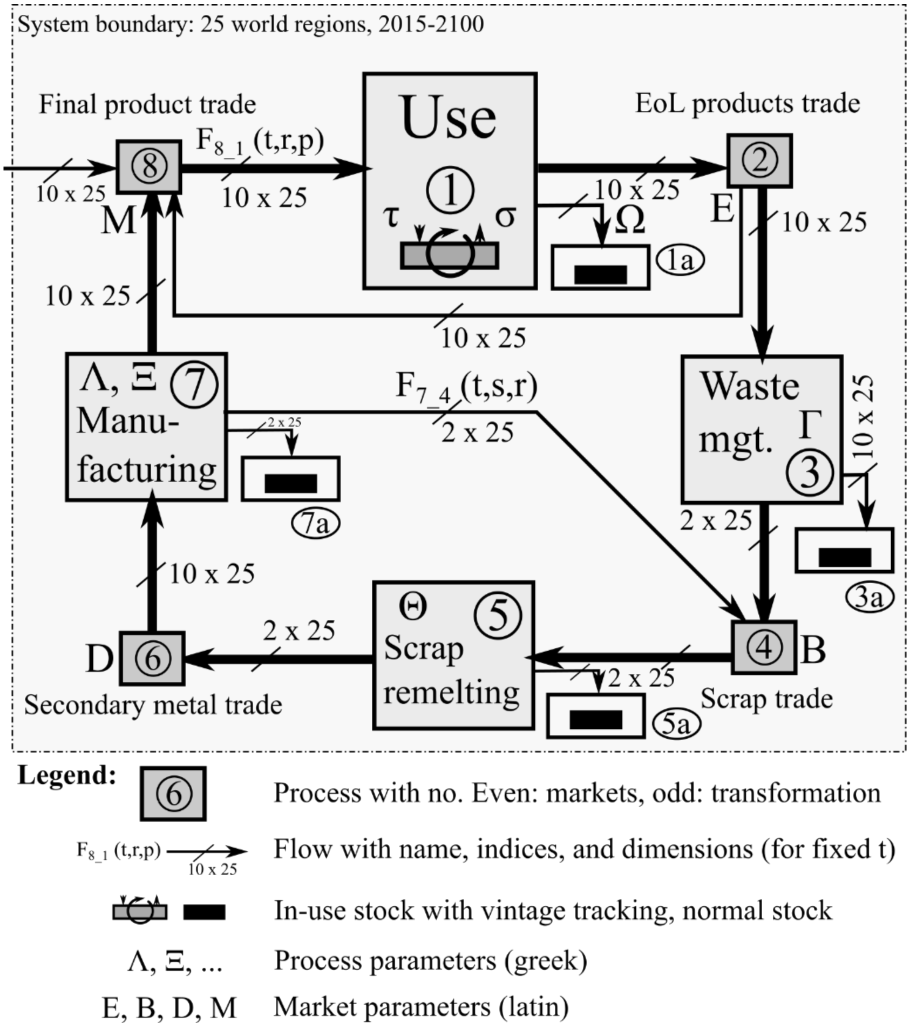

Figure S1: System definition of MaTrace Global.

The indices, system variables, and model parameters of MaTrace Global

Table S1 lists the model indices, the flows, the stocks, and the model parameters.

Table S1: Overview of indices, system variables, and model parameters

Name	Symbol	Size	Explanation
**Indices (Sets)**			
Model time	t	86 x 1	Vector of model time, [2015-2100]
Age cohort	t'	86 x 1	Vector of age cohorts, [2015-2100]
Region	r	25 x 1	Vector of regions, cf. Table S2
Products	p	10 x 1	Vector of products, cf. Table S3
Scrap	s	2 x 1	Vector of scrap types, cf. Table S4
Materials	m	2 x 1	Vector of remelting routes / secondary materials, cf. Table S5
**System variables: Flows.**			
Exogenous input	$F_{\_0\_8}$(t,r,p)	86 x 25 x 10	Original feed of product to be traced, e.g., consumption of passenger car in 2015
Final consumption	$F_{\_8\_1}$(t,r,p)	86 x 25 x 10	Products entering the use phase
EoL products	$F_{\_1\_2}$(t,r,p)	86 x 25 x 10	Products entering markets for EoL products
Addition to obsolete stocks	$F_{\_1\_1a}$(t,r,p)	86 x 25 x 10	Products leaving the use phase and accumulating as obsolete stocks
Re-use of products	$F_{\_2\_8}$(t,r,p)	86 x 25 x 10	Eol products being traded to other regions for re-use
Postconsumer products for treatment	$F_{_2_3}$t,r,p)	86 x 25 x 10	EoL products entering the waste management industries
Landfilled postconsumer waste	$F_{\_3\_3a}$(t,r,p)	86 x 25 x 10	Metal in non-recovered waste fractions, sent to landfill
Postconsumer scrap	$F_{\_3\_4}$(t,r,s)	86 x 25 x 2	Postconsumer scrap for trade
Fabrication scrap	$F_{\_7\_4}$(t,r,s)	86 x 25 x 2	Fabrication scrap for trade
Scrap consumption	$F_{\_4\_5}$(t,r,m)	86 x 25 x 2	Scrap entering the remelting industries
Remelting losses	$F_{\_5\_5a}$(t,r,m)	86 x 25 x 2	Metal lost to the slag phase during remelting
Secondary metal output	$F_{\_5\_6}$(t,r,m)	86 x 25 x 2	Output of secondary metal by refinement process and region
Secondary metal consumption	$F_{\_6\_7}$(t,r,p)	86 x 25 x 10	Secondary metal entering the manufacturing sectors, by region
Fabrication loss	$F_{\_7\_7a}$(t,r,s)	86 x 25 x 2	Fabrication yield loss that is not recovered as scrap and landfilled
Output of manufactured products	$F_{\_7\_8}$(t,r,p)	86 x 25 x 10	Manufactured products sent to market

**System variables: Stocks.**			
In-use stocks	$S_{\_1}(t,t',r,p)$	86 x 86 x 25 x 10	Stock of metal in products in the use phase, by region and product, with vintage tracking
Stock of obsolete products	$S_{\_1a}(t,r,p)$	86 x 25 x 10	Stock of metal in obsolete stocks, such as abandoned buildings or non-recoverable infrastructure
Stock of postconsumer losses in landfills	$S_{\_3a}(t,r,p)$	86 x 25 x 10	Stock of metal in discarded products that was not recovered in the waste management industries
Stock in slag piles	$S_{\_5a}(t,r,m)$	86 x 25 x 2	Stock of metal lost during remelting
Stock of fabrication loss in landfills	$S_{\_7a}(t,r,s)$	86 x 25 x 2	Stock of metal in fabrication scrap that was not recovered
**Model parameters.**			
Product lifetime	$\tau(r,p)$	25 x 10	Mean lifetime by product and region
Standard deviation of product lifetime	$\sigma(r,p)$	25 x 10	Standard deviation of the product lifetime by product and region
Obsolete stock formation rate	$\Omega(t,r,p)$	86 x 25 x 10	Denotes the fraction of the products leaving the use phase in a given year that accumulates as obsolete stocks
Trade of EoL products	$E(t,r,r',p)$	86 x 25 x 25 x 10	E(t,r,r',p) denotes the share of EoL products in year t, product group p, and region r that is sent to region r' for treatment.
EoL recovery rate	$\Gamma(t,r,p,s)$	86 x 25 x 10 x 2	Share of steel in product p, region r, and year t that is recovered as scrap type s
Scrap allocation	$B(t,s,r,m,r')$	86 x 2 x 25 x 2 x 25	Share of scrap of type s in region r in year t that is send to remelting process m in region r'.
Remelting yield	$\Theta(t,m,r)$	86 x 2 x 25	Yield of remelting process m in region r in year t, denotes the fraction of the steel that is transformed into secondary metal.
Sector split	$D(t,m,r,p,r')$	86 x 2 x 25 x 10 x 25	For each year t, D gives the share of metal m in region r that is sent to the manufacturing sectors p in region r'. D thus both allocates secondary metal to manufacturing sectors and contains information on the trade of metal between regions.
Fabrication yield	$\Lambda(t,r,p)$	86 x 25 x 10	Fabrication yield by manufacturing sector p and region r.
Fabrication yield loss recovery	$\Xi(t,r,p,s)$	86 x 25 x 10 x s	Denotes the fraction of the yield loss in manufacturing sector p in region r that gets recovered as scrap type s in year t.
Trade of final products	$M(t,r,r',p)$	86 x 25 x 25 x 10	Share of product p in region r that gets traded to region r' in year t.

Table S2: Model regions

Canada,<br>US,<br>Mexico,<br>Brazil,<br>South Africa,<br>Other Western Europe,<br>Other Central Europe,<br>Russia,<br>RoW Middle East,<br>Turkey,<br>India,<br>Korea,<br>China,<br>Indonesia,<br>Japan,<br>Oceania,<br>RoW America,<br>Row Europe,<br>Row Asia/Oceania,<br>Row Africa,<br>Germany,<br>France,<br>Italy,<br>Spain,<br>UK

Table S3: Product groups

Fabricated metal products, except machinery and equipment<br>Machinery and equipment n.e.c.<br>Office machinery and computers<br>Electrical machinery and apparatus n.e.c.<br>Radio, television and communication equipment and apparatus<br>Medical, precision and optical instruments, watches and clocks<br>Motor vehicles, trailers and semi-trailers<br>Other transport equipment<br>Furniture and other manufactured goods n.e.c.<br>Construction work/buildings

Table S4: Scrap types

Fabrication scrap (new srap)<br>Postconsumer scrap (old scrap)

Table S5: Remelting processes

BOF route (scrap is added to BOF melt)
EAF route

The model equations of MaTrace Global

Below the model equations of MaTrace Global are described. The equations are programmed as Python script, and this script also reads the parameter values from the Excel data file. The model is solved recursively in calculation steps, one for each year, starting in 2015 and terminating in 2100. We first describe the dynamic stock model of the use phase, the only part of the model with vintage tracking, where inputs of previous years determine the outflows of obsolete products in the current model year. Then we describe the equation system for the recycling and reuse loop.

The dynamic stock model for the use phase determines the outflow of products as a consequence of previous consumption and gradual obsolescence. With a normally distributed lifetime assumed, this aging process is commonly described as convolution operation, where the inflow is convoluted (*-operator) with the probability density function *pdf(τ,σ)* for the event that the product leaves the stock (van der Voet, Kleijn, Huele, Ishikawa, & Verkuijlen, 2002):

$$pdf\,(\mathrm{t},\mathrm{t'},\mathrm{r},\mathrm{p};\tau(r,p),\sigma(r,p)) = \frac{1}{\sqrt{2\pi}\cdot\sigma}\cdot e^{\frac{(t-t'-\tau)^2}{2\sigma^2}} \quad (1)$$

Equation 1 shows the pdf for a normal distribution of the lifetime, which has been applied in previous work (Pauliuk, Dhaniati, & Müller, 2012; Pauliuk, Milford, Müller, & Allwood, 2013; Pauliuk, Wang, & Müller, 2012) and which is also used in MaTrace Global. The outflow from the in-use stock $F_y(r,p)$, which is not shown in Figure S1, in a given year is then shown in equation 2, where * represents the convolution:

$$F_y\left(r,p\right) = F_{8-1}\left(r,p\right) * pdf\,(\mathrm{r},\mathrm{p}) \quad (2)$$

A certain fraction (F_1_1a) accumulates as obsolete stocks, another fraction (F_2_8) is traded and re-used, and the remainder (F_2_3) is sent to the domestic waste management industries:

$$F_{1\_1\mathrm{a}}\left(t,r,p\right) = \Omega(t,r,p)\odot F_y\left(t,r,p\right)$$

$$F_{1\_2}\left(t,r,p\right) = (J_{rp}-\Omega(t,r,p))\odot F_y\left(t,r,p\right) \quad (3)$$

$$F_{2\_8}\left(t,r',p\right)=\sum_{r}E(t,r,r',p)\cdot F_{y}\left(t,r,p\right)$$

$$F_{2\_3}\left(t,r,p\right)=(J_{rp}-\Omega-\sum_{r'}E(\ldots,r'))\odot F_{y}\left(t,r,p\right) \quad (4)$$

Where $\odot$ denotes the operator for element-wise multiplication and J_{rp} stands for the unit matrix of size r x p where every single element equals 1.

Next, we use the old scrap recovery parameter Γ to determine both the losses in recovery and the total available old scrap by region, $F_{\_3\_4}$(t,<r,s>):

$$F_{3\_4}\left(t,\langle s,r\rangle\right)=\sum_{p}\Gamma\left(t,r,p,s\right)\cdot F_{2\_3}\left(t,r,p\right) \quad (5)$$

$$F_{3\_3a}\left(t,r,\mathrm{p}\right)=\sum_{s}\left(1-\Gamma\left(t,r,p,s\right)\right)\cdot F_{2\_3}\left(t,r,p\right) \quad (6)$$

The notation F_3_4(t,<s,r>) indicates that for every year t, we store F_3_4 as column vector with outer index s and inner index r.

By solving the dynamic stock model for a given year, we can determine both the re-used products F_2_8 and the recovered postconsumer scrap F_3_4. We now write down the model equations for the recycling loop that comprises the processes 4-7.

For now, we assume that we know the recovered fabrication scrap F_7_4(t,<s,r>), also written as column vector and not as matrix. We can then map the total available scrap to the treatment processes in different regions, using the allocation matrix B(<m,r'>,<s,r>). Here, B is a square matrix with outer row index m (recycling process) and inner row index r' (destination region), and outer column index s (scrap type) and inner column index r (origin region).

$$F_{4\_5}\left(t,\langle m,r'\rangle\right)=B\left(t,\langle m,r'\rangle,\langle s,r\rangle\right)\cdot\left(F_{3\_4}\left(t,\langle s,r\rangle\right)+F_{7\_4}\left(t,\langle s,r\rangle\right)\right) \quad (7)$$

The recovered material F_5_6(t,<m,r>) is

$$F_{5\_6}\left(t,\langle m,r\rangle\right)=\Theta\left(t,\langle m,r\rangle,\langle m,r\rangle\right)\cdot B\left(t,\langle m,r\rangle,\langle s,r'\rangle\right)\cdot F_{4\_5}\left(t,\langle s,r'\rangle\right) \quad (8)$$

Consumption of recovered material by manufacturing sector is

$$F_{6\_7}\left(t,\langle m,r'\rangle\right)=D(t,\langle r',p\rangle,\langle m,r\rangle)\cdot F_{5\_6}\left(t,\langle m,r\rangle\right) \quad (9)$$

Which gives us the total useful output F_7_8 and the equation for F_7_4:

$$F_{7\_8}\left(t,\langle r,p\rangle\right)=\Lambda(t,\langle r,p\rangle,\langle r,p\rangle)\cdot F_{6\_7}\left(t,\langle r,p\rangle\right) \quad (10)$$

$$F_{7\_4}\left(t,\langle s,r\rangle\right)=\Xi(t,\langle s,r\rangle,\langle r,p\rangle)\cdot\left(I-\Lambda(t,\langle r,p\rangle,\langle r,p\rangle)\right)\cdot F_{6\_7}\left(t,\langle r,p\rangle\right) \quad (11)$$

The above equations can be resolved for F, which gives us the equation below (without indices), from which all other system variables in the recycling loop can be calculated using the above equations.

$$F_{7\_4}=\left(I-\Xi\cdot\left(I-\Lambda\right)\cdot D\cdot\Theta\cdot B\right)^{-1}\Xi\cdot\left(I-\Lambda\right)\cdot D\cdot\Theta\cdot B\cdot F_{3\_4} \quad (12)$$

With the market share matrix that distributes the new manufactured goods across the 25 regions, the market balance for process 8 can be established and the vector of final consumption can be computed.

$$F_{\_8\_1}(t,\langle r,p\rangle)=M(t,\langle r,p\rangle,\langle r',p\rangle)\cdot F_{\_7\_8}(t,\langle r',p\rangle)+F_{\_0\_8}(t,\langle r,p\rangle)+F_{\_2\_8}(t,\langle r,p\rangle) \quad (13)$$

With the computation of F_8_1 the model solution for the current year is complete and the script moves on to the next year t + 1, starting again with equation 2.

The complete model, a standalone python script controlled by settings specified in an Excel workbook that also contains the data, is available as supplementary material. The model is described in the section 'Guide to the MaTrace Global model in Python'.

The script performs a mass balance check for each of the eight processes and for the system as a whole. The results of the mass balance checks are reported in the log file.

DATA SOURCES AND MODEL PARAMETERS

We list the data sources and describe each of the 11 model parameters of MaTrace global.

Mean lifetime, τ(r,p)

Location on Data sheet 'Parameter_MaTrace': Columns D-N. Here: Table S6.

In line with previous work (Pauliuk, Dhaniati, et al., 2012; Pauliuk et al., 2013; Pauliuk, Wang, et al., 2012), we assumed a normally distributed lifetime. Mean values for the lifetime of the ten different product groups for the 25 model regions were taken from Pauliuk et al. (2013), who study 10 world regions and 4 product groups, and matched to the MaTrace Global product and region classification. In addition, we assumed that the lifetime of other transportation equipment is 1.5 the lifetime of passenger vehicles, that the lifetime of office machinery is ten years, and the lifetime of other machinery 20 years. For Japan, some specific estimates could be obtained from http://www.nies.go.jp/lifespan/ (Murakami, Oguchi, Tasaki, Daigo, & Hashimoto, 2010). We assumed that the lifetime does not change over time.

Alternative parameter value: For the sensitivity and scenario analysis, the parameter τ can be increased by 30%. This option can be selected in column O of the 'Scenario_Overview' sheet of the Excel data file.

Standard deviation of mean lifetime, σ(r,p)

In line with previous work (Pauliuk, Dhaniati, et al., 2012; Pauliuk et al., 2013; Pauliuk, Wang, et al., 2012), we assumed the standard deviation to be 0.3 of the mean. This value was deduced earlier from Müller et al. (2007). σ changes if τ is increased by 30%. We assumed that the standard deviation of the mean lifetime does not change over time.

Table S6: Mean product lifetime by region, in years.

Years (yr)	Fabricated metal products, except machinery and equipment	Machinery and equipment n.e.c.	Office machinery and computers	Electrical machinery and apparatus n.e.c.	Radio, television and communication equipment and apparatus	Medical, precision and optical instruments, watches and clocks	Motor vehicles, trailers and semi-trailers	Other transport equipment	Furniture; other manufactured goods n.e.c.	Construction work
Canada	15	30	10	20	15	15	20	30	15	75
US	15	30	10	20	15	15	20	30	15	75
Mexico	15	30	10	20	15	15	20	30	15	75
Brazil	15	30	10	20	15	15	20	30	15	75
South Africa	15	30	10	20	15	15	20	30	15	75
Other W Eur.	15	30	10	20	15	15	20	30	15	75
Other C Eur.	15	30	10	20	15	15	20	30	15	75
Russia	15	30	10	20	15	15	20	30	15	75
RoW M.East	15	30	10	20	15	15	20	30	15	75
Turkey	15	30	10	20	15	15	20	30	15	75
India	15	30	10	20	15	15	20	30	15	75
Korea	10	20	10	20	10	10	13	20	10	50
China	15	30	10	20	15	15	20	30	15	75
Indonesia	15	30	10	20	15	15	20	30	15	75
Japan	**15**	20	10	**14.4**	**13.4**	15	**15.6**	23	**13**	**45**
Oceania	10	20	10	20	10	10	13	20	10	50
RoW America	15	30	10	20	15	15	20	30	15	75
Row Europe	15	30	10	20	15	15	20	30	15	75
Row Asia/Oc	15	30	10	20	15	15	20	30	15	75
Row Africa	15	30	10	20	15	15	20	30	15	75
Germany	15	30	10	20	15	15	20	30	15	75
France	15	30	10	20	15	15	20	30	15	75
Italy	15	30	10	20	15	15	20	30	15	75
Spain	15	30	10	20	15	15	20	30	15	75
UK	15	30	10	20	15	15	20	30	15	75

Obsolete stock formation rate, Ω(t,r,p)

Location on Data sheet 'Parameter_MaTrace': Columns Q-AO. Here: Table S7.

Wherever possible, rates for the formation of obsolete stocks, which are highly uncertain and often subject of speculations, were taken from previous work (Nakamura et al., 2014; Pauliuk et al., 2013). For the end-of-life-fate of passenger vehicles in Japan, Germany, and the US, we needed better data based on vehicle reregistration, trade, and scrapping statistics.

Data on EoF vehicle trade for **Japan** were obtained from this web page (last access date 2016-04-09), based on data from the Japanese Ministry of Finance:

http://japan-used-car-exporting.info/import/statistics-export-import-japanese-used-vehicles.html

No data on the amount of deregistered vehicles could be found, and we use the model results of Kagawa et al. (2015) as proxy Since Kagawa et al. do not report obsolete stock formation, we assumed that all deregistered vehicles are either exported or scrapped.

Data for the export of EoL vehicles from the **US** were obtained from this web page (last access date 2016-04-09), based on data from the International Trade Administration:

http://trade.gov/mas/manufacturing/OAAI/tg_oaai_005282.asp

Data for deregistration of vehicles in the US were obtained from this web page (last access date 2016-04-09):

http://www.statista.com/statistics/274931/number-of-vehicles-scrapped-in-the-us/

Since neither source reports obsolete stock formation, we assumed that all deregistered vehicles are either exported or scrapped.

Data on the fate of EoL vehicles in **Germany** were obtained from two different sources (last access date 2016-04-09). 2009 was the latest year for which data were available.

https://www.umweltbundesamt.de/sites/default/files/medien/421/dokumente/abfall-ressourcen_produktverantwortung_altfahrzeuge_vortrag-ihk.pdf

http://www.autogenau.de/news/aktuelles/auto-export-das-statistische-bundesamt-informiert.html

These sources mention a gap of 290000 deregistered vehicles which were not recorded, and we assumed, that this share of 9% of the deregistered vehicles piled up as obsolete stocks.

The parameter Ω is fixed and cannot be changed in the scenario and sensitivity settings. We assumed that the shares for obsolete stock formation do not change over time.

Table S7: Share Ω of end-of-life products going to obsolete stocks. Data sources: light blue shade: (Pauliuk et al., 2013), medium blue shade: (Nakamura et al., 2014), light red: cf. text.

	Products	Machinery and equipment	Office machinery	Electrical machinery	Radio, television and comm.	Medical, precision , optical	Motor vehicles, trailers and semi-trailers	Other transport equipment	Furniture etc	Construction work
Canada	0.08	0.05	0.08	0.05	0.08	0.08	0.01	0.01	0.08	0.10
US	0.08	0.05	0.08	0.05	0.08	0.08	0.00	0.00	0.08	0.10
Mexico	0.08	0.05	0.08	0.05	0.08	0.08	0.01	0.01	0.08	0.10
Brazil	0.08	0.05	0.08	0.05	0.08	0.08	0.01	0.01	0.08	0.10
South Africa	0.08	0.05	0.08	0.05	0.08	0.08	0.01	0.01	0.08	0.10
Other W Eur.	0.08	0.05	0.08	0.05	0.08	0.08	0.01	0.01	0.08	0.10
Other C Europe	0.08	0.05	0.08	0.05	0.08	0.08	0.01	0.01	0.08	0.10
Russia	0.08	0.05	0.08	0.05	0.08	0.08	0.01	0.01	0.08	0.10
RoW M.East	0.08	0.05	0.08	0.05	0.08	0.08	0.01	0.01	0.08	0.10
Turkey	0.08	0.05	0.08	0.05	0.08	0.08	0.01	0.01	0.08	0.10
India	0.08	0.05	0.08	0.05	0.08	0.08	0.01	0.01	0.08	0.10
Korea	0.08	0.05	0.08	0.05	0.08	0.08	0.01	0.01	0.08	0.10
China	0.08	0.05	0.08	0.05	0.08	0.08	0.01	0.01	0.08	0.10
Indonesia	0.08	0.05	0.08	0.05	0.08	0.08	0.01	0.01	0.08	0.10
Japan	0.08	0.05	0.08	0.05	0.08	0.08	0.00	0.00	0.08	0.30
Oceania	0.08	0.05	0.08	0.05	0.08	0.08	0.01	0.01	0.08	0.10
RoW America	0.08	0.05	0.08	0.05	0.08	0.08	0.01	0.01	0.08	0.10
Row Europe	0.08	0.05	0.08	0.05	0.08	0.08	0.01	0.01	0.08	0.10
Row Asia/Oc	0.08	0.05	0.08	0.05	0.08	0.08	0.01	0.01	0.08	0.10
Row Africa	0.08	0.05	0.08	0.05	0.08	0.08	0.01	0.01	0.08	0.10
Germany	0.08	0.05	0.08	0.05	0.08	0.08	0.09	0.09	0.08	0.10
France	0.08	0.05	0.08	0.05	0.08	0.08	0.01	0.01	0.08	0.10
Italy	0.08	0.05	0.08	0.05	0.08	0.08	0.01	0.01	0.08	0.10
Spain	0.08	0.05	0.08	0.05	0.08	0.08	0.01	0.01	0.08	0.10
UK	0.08	0.05	0.08	0.05	0.08	0.08	0.01	0.01	0.08	0.10

Trade of EoL products, E(t,r,r',p)

Location on Data sheet 'Parameter_MaTrace': Columns Q-AO. Here: Table S8.

Trade of EoL products was considered only for EoL vehicles from the three countries Germany, Japan, and the US. The data sources are the same as for the obsolete stock formation rate.

The balance of the flow of deregistered vehicles, 1-Ω-E, is sent to the domestic waste management industries.

The parameter E is fixed and cannot be changed in the scenario and sensitivity settings. We assumed that the trade shares of EoL products do not change over time.

Table S8: Share E of destination regions in the trade of EoL vehicles in Germany, Japan, and the US, respectively.

	Germany	Japan	US
Canada	0.00	0.00	0.00
US	0.00	0.00	0.00
Mexico	0.00	0.00	0.00
Brazil	0.00	0.00	0.00
South Africa	0.00	0.00	0.00
Other W Eur.	0.03	0.01	0.00
Other C Europe	0.24	0.00	0.00
Russia	0.01	0.13	0.00
RoW M.East	0.00	0.02	0.01
Turkey	0.00	0.00	0.00
India	0.00	0.00	0.00
Korea	0.00	0.00	0.00
China	0.00	0.00	0.00
Indonesia	0.00	0.00	0.00
Japan	0.00	0.00	0.00
Oceania	0.00	0.03	0.00
RoW America	0.00	0.03	0.00
Row Europe	0.01	0.00	0.00
Row Asia/Oc	0.01	0.04	0.01
Row Africa	0.03	0.03	0.01
Germany	0.00	0.00	0.00
France	0.01	0.00	0.00
Italy	0.01	0.00	0.00
Spain	0.01	0.00	0.00
UK	0.00	0.01	0.00

Recovery rate of scrap from EoL products, Γ(t,r,p,s)

Location on Data sheet 'Parameter_MaTrace': Columns AQ-BA. Alternative scenario: Columns BC-BM. Here: Table S9.

Data for the current and possible future scrap recovery rate from EoL products were taken from WorldSteel (World Steel Association, 2010). We used these data, which are listed in Table S9, and interpolated them linearly to obtain values for the years between 2008 and 2050. No additional improvements were considered for the time after 2050. The ten categories or MaTraceGlobal were matched to the four categories reported by WorldSteel.

Alternative parameter value: For the sensitivity and scenario analysis, the parameter Γ can be increased to 95% in all categories. This improvement anticipates a significant improvement in the dismantling and sorting processes (Ignatenko, van Schaik, & Reuter, 2008; Reuter, van Schaik, Ignatenko, & de Haan, 2006). This option can be selected in column V of the 'Scenario_Overview' sheet of the Excel data file.

In MaTrace Global, the parameter Γ does change over time.

Table S9: Recovery rates of EoL scrap, reference values

	Vehicles	Industry	Construction	Products
**2008**	0.85	0.9	0.85	0.5
**2050**	0.95	0.95	0.9	0.75
**2050 (alternative)**	0.95	0.95	0.95	0.95

Allocation of scrap to refinement processes and regions, B(t,s,r,m,r')

Location on Data sheet 'Parameter_MaTrace': Columns BO-CN. Here: Table S12.

Parameter B allocates both fabrication and postconsumer scrap to one of the two remelting processes in any other region. It thus combines a trade pattern for scrap with a process selection.

The trade pattern for scrap was determined for the base year 2007, as for this year, both scrap trade date from UN ComTrade (Gaulier & Zignago, 2010) as scrap supply data from EXIOBASE v2 (Wood et al., 2014) were present. For the scrap trade data, trade in the following commodity groups was added together and then aggregated to the 25 regions (Table S10).

Table S10: Commodity groups used for determination of scrap trade shares. HS code number and group name.

**720410**	Waste and scrap of cast iron
**720421**	Waste and scrap Of stainless steel
**720429**	Waste and Scrap of Other Ally Steel
**720430**	Waste and scrap of tinned iron or steel
**720441**	Other waste and scrap:Turnings, shavings, chips, milling waste, sawdust, filings, trimmings and stampings,whether or not in bundles
**720449**	Other Ferrous Waste and Scrap
**720450**	Remelting scrap ingots

The total scrap export from each region was subtracted from the total scrap supply to determine the apparent scrap use. With both domestic use and export flows, the percentages of total scrap supply being exported to the different regions and being remelted domestically were determined (Table S11).

Table S11: Allocation coefficients of scrap supply to other regions (export) and domestically. The table has two parts, the first one shows the trade shares for trade to Canada, US, … and China, and the second one shows the shares for trade to Indonesia, Japan, … and the UK.

Row: From, Column: To	Canada	US	Mexico	Brazil	South Africa	Other W Europe	Other C Europe	Russia	RoW M.East	Turkey	India	Korea	China
Canada	0.30	0.42	0.00	0.00	0.00	0.00	0.01	0.00	0.01	0.07	0.03	0.01	0.06
US	0.04	0.55	0.03	0.00	0.00	0.00	0.01	0.00	0.01	0.10	0.02	0.05	0.03
Mexico	0.00	0.04	0.95	0.00	0.00	0.00	0.00	0.00	0.00	0.00	0.00	0.00	0.00
Brazil	0.00	0.00	0.00	0.99	0.00	0.00	0.00	0.00	0.00	0.00	0.00	0.00	0.00
South Africa	0.00	0.00	0.00	0.00	0.77	0.00	0.00	0.00	0.00	0.00	0.03	0.00	0.00
Other W Eur.	0.01	0.01	0.00	0.00	0.00	0.26	0.05	0.00	0.02	0.17	0.02	0.01	0.01
Other C Eur.	0.00	0.00	0.00	0.00	0.00	0.02	0.55	0.00	0.00	0.14	0.00	0.00	0.00
Russia	0.00	0.00	0.00	0.00	0.00	0.02	0.02	0.60	0.01	0.14	0.00	0.04	0.00
RoW M.East	0.00	0.00	0.00	0.00	0.00	0.00	0.01	0.00	0.71	0.05	0.08	0.00	0.00
Turkey	0.00	0.00	0.00	0.00	0.00	0.00	0.00	0.00	0.00	0.99	0.00	0.00	0.00
India	0.00	0.00	0.00	0.00	0.00	0.00	0.00	0.00	0.00	0.00	1.00	0.00	0.00
Korea	0.00	0.00	0.00	0.00	0.00	0.00	0.00	0.00	0.00	0.00	0.00	0.99	0.00
China	0.00	0.00	0.00	0.00	0.00	0.00	0.00	0.00	0.00	0.00	0.00	0.00	1.00
Indonesia	0.00	0.00	0.00	0.00	0.00	0.00	0.00	0.00	0.00	0.00	0.00	0.00	0.00
Japan	0.00	0.00	0.00	0.00	0.00	0.00	0.00	0.00	0.00	0.00	0.00	0.09	0.05
Oceania	0.00	0.00	0.00	0.00	0.00	0.00	0.00	0.00	0.00	0.00	0.03	0.00	0.07
RoW America	0.00	0.01	0.01	0.00	0.00	0.00	0.00	0.00	0.00	0.00	0.00	0.01	0.02
Row Europe	0.00	0.00	0.00	0.00	0.00	0.01	0.05	0.00	0.02	0.04	0.00	0.00	0.00
Row Asia/Oc	0.00	0.00	0.00	0.00	0.00	0.00	0.01	0.00	0.00	0.02	0.01	0.01	0.04
Row Africa	0.00	0.00	0.00	0.00	0.02	0.00	0.00	0.00	0.01	0.08	0.07	0.00	0.00
Germany	0.00	0.00	0.00	0.00	0.00	0.21	0.03	0.00	0.00	0.02	0.00	0.00	0.00
France	0.00	0.00	0.00	0.00	0.00	0.31	0.00	0.00	0.00	0.01	0.00	0.00	0.00
Italy	0.00	0.00	0.00	0.00	0.00	0.00	0.00	0.00	0.00	0.00	0.00	0.00	0.00
Spain	0.00	0.00	0.00	0.00	0.00	0.01	0.00	0.00	0.00	0.00	0.00	0.00	0.01
UK	0.00	0.02	0.00	0.00	0.00	0.09	0.03	0.00	0.05	0.26	0.02	0.00	0.01

Row: From, Column: To	Indonesia	Japan	Oceania	RoW America	Row Europe	Row Asia/Oc	Row Africa	Germany	France	Italy	Spain	UK
Canada	0.00	0.00	0.00	0.00	0.00	0.07	0.00	0.00	0.00	0.02	0.00	0.00
US	0.01	0.00	0.00	0.01	0.00	0.13	0.00	0.00	0.00	0.00	0.00	0.00
Mexico	0.00	0.00	0.00	0.00	0.00	0.00	0.00	0.00	0.00	0.00	0.00	0.00
Brazil	0.00	0.00	0.00	0.00	0.00	0.00	0.00	0.00	0.00	0.00	0.00	0.00
South Africa	0.01	0.00	0.00	0.00	0.00	0.19	0.00	0.00	0.00	0.00	0.00	0.00
Other W Eur.	0.00	0.00	0.00	0.00	0.00	0.02	0.00	0.21	0.08	0.02	0.10	0.01
Other C Eur.	0.00	0.00	0.00	0.00	0.03	0.00	0.00	0.13	0.00	0.10	0.02	0.00
Russia	0.00	0.00	0.00	0.00	0.07	0.03	0.00	0.00	0.01	0.00	0.05	0.00
RoW M.East	0.01	0.00	0.00	0.00	0.00	0.12	0.00	0.00	0.00	0.00	0.00	0.00
Turkey	0.00	0.00	0.00	0.00	0.00	0.00	0.00	0.00	0.00	0.00	0.00	0.00
India	0.00	0.00	0.00	0.00	0.00	0.00	0.00	0.00	0.00	0.00	0.00	0.00
Korea	0.00	0.00	0.00	0.00	0.00	0.00	0.00	0.00	0.00	0.00	0.00	0.00
China	0.00	0.00	0.00	0.00	0.00	0.00	0.00	0.00	0.00	0.00	0.00	0.00
Indonesia	0.98	0.00	0.00	0.00	0.00	0.01	0.00	0.00	0.00	0.00	0.00	0.00
Japan	0.00	0.84	0.00	0.00	0.00	0.02	0.00	0.00	0.00	0.00	0.00	0.00
Oceania	0.11	0.00	0.38	0.00	0.00	0.40	0.00	0.00	0.00	0.00	0.00	0.00
RoW America	0.01	0.00	0.00	0.84	0.00	0.08	0.00	0.00	0.00	0.00	0.01	0.00
Row Europe	0.00	0.00	0.00	0.00	0.86	0.00	0.00	0.00	0.00	0.02	0.00	0.00
Row Asia/Oc	0.01	0.00	0.00	0.00	0.01	0.87	0.00	0.00	0.00	0.00	0.00	0.00
Row Africa	0.01	0.00	0.00	0.00	0.00	0.07	0.72	0.00	0.00	0.01	0.01	0.00
Germany	0.00	0.00	0.00	0.00	0.00	0.01	0.00	0.57	0.08	0.08	0.01	0.00
France	0.00	0.00	0.00	0.00	0.00	0.00	0.01	0.06	0.26	0.13	0.21	0.00
Italy	0.00	0.00	0.00	0.00	0.00	0.00	0.00	0.00	0.00	0.98	0.01	0.00
Spain	0.00	0.00	0.00	0.00	0.00	0.00	0.00	0.00	0.00	0.00	0.98	0.00
UK	0.01	0.00	0.00	0.00	0.00	0.13	0.00	0.00	0.06	0.00	0.18	0.13

With the matrix in the table S11 called B_0, we then assembled the following two versions of B (Table S12). In the baseline, new scrap is traded to other regions according to the shares in B_0, and assigned to the BOF route, which is the preferred destination of new scrap. Old scrap is traded according to B_0 as well, but assigned to the EAF route, which is the default for postconsumer scrap in many regions.

Alternative parameter value: In the advanced scenario, postconsumer scrap is traded according to B_0 as well, but assigned to the BOF route, which can be considered an advanced case as BOF steel meets tougher specification, and dilution with scrap requires the feed to be of high purity. Since we trace a small material unit only, we can safely assume that BOF production levels are high enough to absorb the scrap considered in the model. The advanced option for B can be selected in column P of the 'Scenario_Overview' sheet of the Excel data file.

We assumed that the lifetime does not change over time.

Table S12: Combination of trade pattern for steel scrap (B_0) into the scrap allocation matrix B.

Baseline				Advanced		
	New scrap	Old Scrap			New scrap	Old Scrap
BOF route	B_0	0		BOF route	B_0	B_0
EAF route	0	B_0		EAF route	0	0

Remelting yield, Θ(t,m,r)

Location on Data sheet 'Parameter_MaTrace': Columns CU-CV.

Some information exists on yield losses in the basic oxygen furnace and the electric arc furnace, but it is not consistent. In the 'Steel scrap age', Pauliuk et al. (2013) estimate a remelting yield for iron of 93.8% for the BOF and 95.7% for the EAF, based on a WorldSteel report (World Steel Association, 2009). A report commissioned by the American Iron and Steel institute finds EAF yields of 92-94% and BOF yields of 91-93%. This report does not specify whether the losses are due to removal of impurities such as paint or whether they are due to metal oxidation. Stubbles points out that in the BOF companion metals like manganese or silicon are oxidized before iron, and that the iron yield losses in the BOF are 3.7%, which means an Fe-yield of 96.3% (Stubbles, n.d.). The AISI also asserts that yield losses should be reduced by 33%.

Based on the spectrum of values we found for the yield losses, we assume an **average iron yield in both EAF and BOF to be 94%**.

Alternative parameter value: To account for the uncertainty of the remelting yield, we propose an **alternative value of 97%**, which we analyze in the sensitivity analysis and which also enters some improvement scenarios. The advanced option for Θ can be selected in column Q of the 'Scenario_Overview' sheet of the Excel data file.

Allocation of secondary metal to products and other regions, D(t,m,r,p,r')

Location on Data sheet 'Parameter_MaTrace': Columns CX-EW.

The matrix D, which is colloquially called 'sector split', is determined from a material concentration matrix C and the final demand vector for steel-containing products y (Nakamura et al., 2014)

$$
\begin{aligned}
D &= \hat{y} \cdot C \cdot \left(y^T \cdot C\right)^{\wedge -1} \\
&= \hat{y} \cdot C \cdot \hat{d}^{-1}, \text{ where } d = y^T \cdot C
\end{aligned} \tag{14}
$$

The final demand vector y is obtained from EXIOBASE v2 and aggregated to the 25 model regions. The concentration array C is determined using the WIO-MFA approach (Nakamura, Nakajima, Kondo, & Nagasaka, 2007) according to equation (15):

$$C^T = \widetilde{A}_{MP} \cdot \left(1 - \widetilde{A}_{PP}\right)^{-1} \quad (15)$$

Where the two A-matrices are submatrices of and MRIO-A-matrix corrected for yield losses (Nakamura et al., 2007; Ohno, Matsubae, Nakajima, Nakamura, & Nagasaka, 2014).

The MRIO-A-matrix used to determine the material concentration was determined as follows:

We developed a multi-regional commodity-by-commodity direct requirement matrix (A) based on EXIOBASE version 2.2 (Wood et al., 2014) by the three steps described below. The main purpose of developing the direct requirement matrix is to estimate matrix D through the WIO-MFA approach. In estimating a symmetric input–output table (IOT), therefore, we combined the byproduct-technology construct (Suh, Weidema, Schmidt, & Heijungs, 2010) and another construct which are suitable for estimating the material composition of products.

Step 1. Scrap generation is recorded as the supply of basic metal products in the supply table. We assumed that the supply of basic metal products by an industry which does not use metal ores is scrap generation, and that if an industry uses metal ores, any supply of basic metal products by that industry is not scrap generation. Table S13 shows the list of basic metal products and metal ores. We selected industries which generated metal scrap on the criterion directly driven by the assumption. The criterion was separately employed for the ferrous metal and the group of all nonferrous metals. Then, we modified the EXIOBASE monetary supply and use tables (SUTs) by applying the byproduct-technology construct to the pairs of the selected industries and basic metal products.

Step 2. We applied a simplified version of the Smith and McDonald method (Smith & McDonald, 2011) to the modified EXIOBASE monetary national SUTs. The objective function of the Smith and McDonald method is a weighted sum of the two quadratic distances. Of the two, we adopted only one distance that is to minimize for obtaining a construct closest to the commodity-technology construct (Suh et al., 2010), on our purpose to estimate the material composition matrix. The non-negativity constraints on variables related to the modification described in Step 1 were relaxed. The constrained least squares problems were successfully solved for 36 regions. For the other 12 regions, we adopted the constrained least absolute deviation problems. Convergence was not achieved for four of the 12 regions. We thus applied the industry-technology construct (Suh et al., 2010) to those regions.

Step 3. We constructed a multi-regional monetary commodity-by-commodity IOT by linking the national symmetric IOT of 48 regions estimated in Step 2. Data on bilateral international trade are available in the EXIOBASE database. It was assumed that exporter-countries' shares of the use of imported commodities are common for all industries in a region.

Table S13: List of basic metal products and metal ores

Basic metal products		Metal ores	
Ferrous metal			
p27.a	Basic iron and steel and of ferro-alloys and first products thereof	p13.1	Iron ores
Nonferrous metals			
p27.41	Precious metals	p13.20.11	Copper ores and concentrates
p27.42	Aluminium and aluminium products	p13.20.12	Nickel ores and concentrates
p27.43	Lead, zinc and tin and products thereof	p13.20.13	Aluminium ores and concentrates
p27.44	Copper products	p13.20.14	Precious metal ores and concentrates
p27.45	Other non-ferrous metal products	p13.20.15	Lead, zinc and tin ores and concentrates
		p13.20.16	Other non-ferrous metal ores and concentrates

With the so-obtained Matrix A, material concentrations C and ultimately D were calculated for average steel, 25 regions, and 10 product groups in the reference year 2007. We used the D matrix to allocate BOF steel to products in different regions. For EAF steel, we assume that the trade shares between regions would be the same as for BOF steel, but all EAF steel is used in construction. Hence, the EAF part of D is obtained from the BOF part by summing up the shares for different products in each destination region and assigning this sum to construction, while setting the shares of all other product groups to zero. A summary of D, here with trade with different regions summed up to make the table fit onto a single page, is shown in table S14.

Alternative parameter value: To account for the difference in trade and application patterns between BOF and EAF steel and possible changes over time, two alternatives for D are given, which are described below. The alternative options for D can be selected in column R of the 'Scenario_Overview' sheet of the Excel data file.

Table S14: D matrix, aggregation, base case. The full table is shown in the Excel data file. The column sum for BOF and EAF steel, respectively, equals 100%

Steel type	Destination	End-use sector	US	Other America	Japan	China	Other Asia/Oceania	Russia	Germany	Other Europe	Middle East	Africa
BOF steel	Domestic use shares	Products/Appliances	6	5	2	3	4	23	6	10	6	6
		Machinery	21	12	18	16	19	6	11	15	16	14
		Computers	0	0	0	1	2	0	0	0	1	0
		Electrical machinery	2	2	2	8	4	0	1	3	3	3
		Communication prods.	1	1	1	1	2	0	0	1	1	1
		Medical & optical products	2	2	1	0	1	0	1	1	1	1
		Cars	29	21	13	7	12	0	15	14	14	20
		Other vehicles	5	2	3	3	3	0	2	3	3	3
		Furnitures	7	2	1	1	3	0	0	2	6	1
		Buildings	20	28	28	42	28	33	4	16	33	17
	Foreign use shares	Products/Appliances	0	2	1	1	1	4	9	3	1	3
		Machinery	1	5	6	3	5	9	13	7	4	7
		Computers	0	0	1	1	1	0	0	0	0	0
		Electrical machinery	0	1	1	1	1	1	2	1	1	1
		Communication prods.	0	0	1	1	1	1	1	1	0	1
		Medical & optical products	0	0	0	0	0	0	1	0	0	0
		Cars	3	7	7	3	6	8	13	9	5	8
		Other vehicles	0	1	2	1	1	1	3	1	1	1
		Furnitures	0	1	1	1	1	3	2	2	1	1
		Buildings	2	7	10	5	8	11	15	11	5	10
EAF steel	Domestic	Buildings	92	75	69	84	75	62	41	64	82	66
	Foreign	Buildings	8	25	31	16	25	38	59	36	18	34

To build an alternative scenario for D that acknowledges the different use shares of BOF and EAF steel, we uses data from the Japanese IO table (Ohno et al., 2015), that distinguishes between EAF and BOF steel, to disaggregate steel flows in the CREEA table into BOF and EAF steel flows. Disaggregation was done by first aggregating the Japanese IOT to the CREEA classification and then using the ratio of BOF and EAF steel flows in the national Japanese table to split the steel flows in the multiregional CREEA table. It was assumed that the split factors are the same in all regions. With the thus disaggregated CREEA table, two allocation matrices, one for BOF and one for EAF steel, were build using equations 14 and 15. A summary of D, which is the concatenation of these to allocation matrices, is shown in table S15. In this table, trade with different regions was summed up to make the table fit onto a single page.

Table S15: D_Matrix, refined with Japanese split between BOF and EAF steel. The column sum for BOF and EAF steel, respectively, equals 100%.

Steel type	Destination	End-use sector	US	Other America	Japan	China	Other Asia	Russia	Germany	Other Europe	Middle East	Africa
BOF steel	Domestic use shares	Products/Appliances	7	5	2	4	4	28	6	11	7	6
		Machinery	21	14	20	20	22	7	12	16	19	15
		Computers	1	0	1	2	2	0	0	0	1	0
		Electrical machinery	2	3	2	10	4	0	1	3	3	4
		Communication	1	1	2	1	3	0	0	1	1	1
		Medical&opt prods	2	2	1	0	1	0	1	1	1	1
		Cars	30	24	14	8	14	0	15	15	16	22
		Other vehicles	5	3	4	4	3	0	2	3	3	3
		Furnitures	8	3	2	1	3	0	0	2	8	1
		Buildings	17	18	22	29	18	22	4	13	22	12
	Foreign use shares	Products/Appliances	0	2	2	1	2	5	9	3	2	4
		Machinery	1	6	7	4	6	11	14	8	4	8
		Computers	0	0	1	1	1	0	0	1	0	1
		Electrical machinery	0	1	1	1	1	2	2	1	1	2
		Communication	0	0	1	1	1	1	1	1	0	1
		Medical&opt prods	0	0	1	0	0	1	1	1	0	0
		Cars	3	8	8	4	7	9	14	9	6	9
		Other vehicles	0	1	2	1	1	2	3	1	1	1
		Furnitures	0	1	1	1	1	3	2	2	1	2
		Buildings	2	6	7	5	7	10	12	8	4	7
EAF steel	Domestic use shares	Products/Appliances	6	3	1	2	2	13	5	8	4	4
		Machinery	19	8	12	9	12	3	10	11	10	10
		Computers	0	0	0	0	0	0	0	0	0	0
		Electrical machinery	1	1	1	3	1	0	1	1	1	1
		Communication	0	0	1	0	1	0	0	0	0	0
		Medical&opt prods	1	1	0	0	0	0	0	0	0	0
		Cars	27	14	9	4	8	0	13	10	9	15
		Other vehicles	4	1	2	2	1	0	2	2	1	2
		Furnitures	4	1	0	0	1	0	0	1	2	1
		Buildings	30	52	43	69	52	56	6	27	58	31
	Foreign use shares	Products/Appliances	0	1	1	1	1	2	7	2	1	2
		Machinery	1	3	4	2	3	5	12	5	2	5
		Computers	0	0	0	0	0	0	0	0	0	0
		Electrical machinery	0	0	1	0	0	1	1	1	0	1
		Communication	0	0	0	0	0	0	0	0	0	0
		Medical&opt prods	0	0	0	0	0	0	1	0	0	0
		Cars	3	5	5	2	4	4	12	7	3	6
		Other vehicles	0	1	1	0	1	1	2	1	0	1
		Furnitures	0	0	0	0	0	1	1	1	0	1
		Buildings	4	9	18	6	11	15	26	22	7	17

To test how D might change over time as final demand in different world region growths, we built a time series for y and recalculated D with a new y for each model year. The calculations are performed by the Python script. The scenario for GDP growth was taken from the OECD database (https://data.oecd.org/gdp/real-gdp-forecast.htm), aggregated to the 25 model regions, and transformed into annual growth rates.

Data for the concentration matrix C for BOF and EAF steel are located on the data sheet 'Parameter_MaTrace': Columns HC-IZ.

Data for the GDP growth rates are located on the data sheet 'Parameter_MaTrace': Columns JD-KC.

Fabrication yield, Λ(t,r,p)

Location on Data sheet 'Parameter_MaTrace': Columns EY-FI.

Data on fabrication yield for steel are available as average global estimates (Cullen, Allwood, & Bambach, 2012) (Table S16). We mapped these yield factors to the 10 product groups in MaTrace Global and used them as based case for all regions and all future years.

Table S16: Fabrication yield rates, reference/base case and possible best practice for2050.

**Base case (2007)**	Vehicles	Industry	Construction	Products
Global	0.733	0.829	0.926	0.773
**Best case (2050)**				
Global	0.81	0.88	0.93	0.85

Alternative parameter value: Fabrication yield improvement is a central material efficiency strategy (Allwood et al., 2012; Milford, Pauliuk, Allwood, & Müller, 2013). The latter article cited gives an estimate of possible yield rates in 2050, and we used these values to construct an alternative scenario for Λ. Values for the years between 2007 and 2050 were obtained via linear interpolation. The alternative options for Λ can be selected in column T of the 'Scenario_Overview' sheet of the Excel data file.

Fabrication scrap recovery rate, Ξ(t,r,p,s)

Location on Data sheet 'Parameter_MaTrace': Columns FK-FU.

The values for the recovery rate of yield loss in fabrication were taken directly from the original MaTrace paper (Nakamura et al., 2014), in which the Year Book of Raw Ferrous Materials 2010 issued by the Japan Ferrous Raw Materials Association is cited. We mapped these yield loss recovery factors to the 10 product groups in MaTrace Global and used them as based case for all regions and all future years.

Table S17: Fabrication yield loss recovery rates, reference/base case and possible best practice for2050.

**Base case (2010)**	Vehicles	Industry	Construction	Products
Global	0.8	0.9	0.7	0.75
**Best case (2050)**				
Global	0.95	0.95	0.95	0.95

Alternative parameter value: Improvements in the recovery of fabrication yield losses are part of the spectrum of material efficiency strategies (Allwood et al., 2012; Milford et al., 2013). To study the impact of increase recovery rates we build an alternative scenario for Ξ by assuming in 2050, 95% of all yield losses would be recovered. Values for the years between 2007 and 2050 were obtained via linear interpolation. The alternative options for Ξ can be selected in column U of the 'Scenario_Overview' sheet of the Excel data file.

Trade of final products, M(t,r,r',p)

Location on Data sheet 'Parameter_MaTrace': Columns FW-GW.

The allocation of fabricated metal products to consuming regions was obtained from extracting the balanced flows on production, trade, and consumption of products from the EXIOBASE v2 supply and use table, aggregating them to the 25 regions and 10 products of MaTrace Global, and determining the allocation coefficients by dividing the flows of each commodity by its total supply. Thus, M is a Ghosh-type matrix of market allocation coefficients (Miller & Blair, 2009).

THE CIRCULARITY PERFORMANCE METRIC Circ(t):

Rationale:

Environmental and resource policy increasingly relies on quantitative indicators for which targets or caps are defined. The 2°C or 1.5°C target for climate change mitigation are prominent examples. The circular economy concept has gained much attention recently, but specific circularity indicators are largely lacking, as is a debate of the nature and coverage of such indicators. The recently proposed Material Circularity Indicator (Ellen MacArthur Foundation, 2015) takes a static perspective on material and product cycles, thus neglecting the time delay between metal consumption and scrap supply due to product lifetime as well as the interlinkages between different product life cycles as materials contained in one product get recycled and used in another product. As MaTrace offers a specific perspective on socioeconomic metabolism, which is complementary to the perspectives taken by life cycle assessment and material flow analysis, we propose to use the results generated by MaTrace to derive a circularity indicator that refers to a specific amount of material consumed at a point in time t_0. The purpose of this indicator is to provide a relative measure of the service provided by this material unit over a certain time period, it can thus be seen as circularity indicator of a unit of material through different product life cycles. It provides an alternative perspective on material cycles compared to the different recycling metrics defined by Graedel et al. (2011), which describe an entire material cycle at a given point in time, and the circularity index for a product throughout its lifecycle, which takes a life cycle perspective for a single product consisting of multiple materials (whole product approach) or single materials (comprehensive approach) .(Ellen MacArthur Foundation, 2015).

Future research will show how the information provided by the three different indicator families differ, what specific research, design, and management questions the indicators can help to answer, and whether and how they could become part of specific policies.

Definition of the circularity indicator for a unit of material through different product lifecycles:

We focus on a single material, here steel, and first define a measure for the cumulative service provided by a unit of material over time. While the physical services provided by different products differ substantially and cannot be directly compared on a physical basis (shelter for buildings, transport for cars, etc.), the services that a specific material provides can often be reduced to a few categories. In the example of copper, the three categories electrical conductivity, heat conductivity, and corrosion/weathering protection cover most of the applications of this metal. In the case of steel the most dominant application by far is structural stability. For the circularity of a metal cycle, however, it is not so much the service provided that is relevant for the indicator but the purity, quality, and recoverability of the metal. For the circularity indicator we can therefore propose to simply use the mass of the material in its different applications and sinks (denoted by the shares of the material in these stocks, x_U), weighted by a factor w that measures purity, quality, and recoverability ($0 \leq w \leq 1$). The share of the material that is present in form of highest quality and highly recoverable applications is assigned $w = 1$, whereas losses in landfills or slag piles would be weighted at $w = 0$. With these specifications we define the useful mass m_U of a certain amount of steel m_0 in its different applications i at a time t as

$$m_U(t) = m_0 \cdot \sum_i x_{U\_i}(t) \cdot w_i = m_0 \cdot x_U(t) \bullet w \qquad (16)$$

where the big dot denotes the summation over the application index *i*. We can then define the cumulative useful mass CUM(T) over a time interval $t_0 \leq t \leq T$ as

$$CUM(T) = m_0 \cdot \int_{t_0}^{T} \left(x_U(t) \bullet w \right) \cdot dt \qquad (17)$$

CUM(T) is measured in *kg*yr*. It is a measure of the availability of the material for high quality applications during a given time period, and thus also a measure of the potential useful service that unit of material can provide during that certain time period. The maximal cumulative useful mass of a kg of high quality material is equal to CUM(T) = 1kg * (T- t_0), and this situation would occur if no losses and down-cycling were observed during the entire calculation period.

Finally, we define the circularity index Circ(T) as a relative measure of the cumulative useful mass provided by the material in terms of the CUM of the ideal case:

$$Circ(T) = \frac{CUM(T)}{m_0 \cdot (T - t_0)} \qquad (18)$$

$$Circ(T) = \frac{1}{(T - t_0)} \cdot \int_{t_0}^{T} \left(x_U \bullet w \right) \cdot dt$$

Circ(T) depends on the development of the different application shares x_U over time; it is independent of the actual mass of metal in the system. Similar to the global warming potential (GWP), Circ(T) depends on the time horizon *T*. In the paper we calculate Circ(2100) for 1 ton of primary steel consumed in 2015 in different applications in different regions for different future scenario of metal recovery, recycling, and international trade.

Consideration of different material qualities:

While MaTrace global is used to calculate the different use shares of the material (x_U) over time, the quality weighting factors *w* needs to be specified as an additional input to the calculation.

The simplest way of defining *w* is to consider all steel in the use phase with weight 1 and all steel/iron that is lost with 0:

$$w = \begin{cases} 1, & \text{use phase} \\ 0, & \text{losses} \end{cases} \qquad (19)$$

The Circ(2100) indicator calculated in this paper uses the simplest version of the weighting as shown in equation 19. This approach does not require us to make subjective assumptions on the relative weights of the different material qualities, which is why we use it in the paper. It does, however, not include any penalty for low-quality recycling, which is why a discussion on meaningful weighting of lower-quality applications is needed. The Circ(2100) indicators calculated with equation 19 thus represent upper limits of a spectrum of possible circularity indicators for a unit of material in different life cycles.

In a next step, one could distinguish between use in high-quality applications including vehicles, machines, and appliances, denoted as case (a), applications of lower quality, here construction steel, case (b), and losses at the different stages of the system (c). One would then set the high-quality applications as reference and define w[a] = 1, set w[c] = 0, and set w[b] = x, where x is the relative weight of the down-cycling application in terms of the original applications (a). The choice of x is to a large extent subjective. The choice of subjective weighting factors is common in life cycle assessment, especially for the determination of endpoint indicators in life cycle impact assessment. In most material flow analysis-related research weighting is not necessary. As soon as performance indicators for materials or material cycles are to be derived, weighting may become necessary also in MFA, however. A good example for weighting in MFA is the criticality metrics developed by Graedel et al. (Graedel et al., 2012; Graedel, Harper, Nassar, Nuss, & Reck, 2015).

For a more differentiated determination of the Circ indicator the weighting factors could be derived in an objective manner directly from the physical properties of the material in its different applications. Possible properties include: the main metal content (e.g., for copper), the yield strength, (for steel), the tramp metal content (for steel and aluminium), or the ductility (for aluminium). Eligible properties need to be evaluated by experts for the recycling of the different metals.

SCENARIO DEFINITION

The above described parameter variations can be studied in isolation from each other (sensitivity analysis) or combined to form scenarios for possible future steel cycles. We first list the different options for parameter changes and then define the scenarios tested.

Sensitivity analysis

We test the sensitivity for changes in 7 of the 11 model parameters. An independent change of σ is not considered. Next to the parameter changes discussed above, we also investigate the consequences of natural declines in global trade or forced trade restrictions in all four markets. Table S18 lists the variations studied.

Table S18: Sensitivity analysis, overview.

Number	Name	Parameter	Description of change (for details see above)
**1**	S_lifetime	Lifetime τ	Increase of mean lifetime and its standard deviation by 30%.
**2**	S_BOF_route	Scrap trade B	Divert postconsumer scrap from EAF into BOF route, trade pattern remains unchanged.
**3**	S_SectorSplit_Static	Sector split D	Use the alternative sector split that was calculated including the Japanese IO data on BOF and EAF steel use
**4**	S_SectorSplit_Dynamic	Sector split D	Use alternative sector split as above, included changes in y due to economic growth in the calculation
**5**	S_fab_yield	Fabrication yield Λ	Gradual increase to 95% in all manufacturing sectors
**6**	S_yield_loss_recov.	Fabrication yield loss recovery Ξ	Gradual increase to 95% in all manufacturing sectors
**7**	S_EoL_recovery	Postconsumer scrap recovery Γ	Gradual increase to 95% for all product types
**8**	S_RemeltingYield	Remelting yield Θ	Increase from 94 to 97%.
**9**	S_no_trade	Market parameters E, B, D, and M	Collapse trade in all trade-containing parameters so that all incoming flows are sent to domestic industries. Allocation shares onto different target industry types (e.g. manufacturing sectors) remain unaffected.
**10**	S_no_EoL_trade	Eol product trade E	Collapse trade as described above, for E only
**11**	S_no_Scrap_trade	Scrap trade B	Collapse trade as described above, for B only
**12**	S_no_Metal_trade	Metal trade D	Collapse trade as described above, for D only
**13**	S_no_Product_trade	Product trade M	Collapse trade as described above, for M only

Scenarios for improving steel cycle circularity.

Table S19 lists the definition of the scenarios studied, and Table S20 list the storylines behind these scenarios.

Table S19: Definition of scenarios.

Scenario name	Parameter description
**Baseline**	Baseline values for all parameters
**HighRecovery**	Baseline + improvements in fabrication yield, yield loss recovery, and postconsumer scrap recovery as well as a higher remelting yield
**HighRecovery_lifetime**	HighRecovery scenario + prolonged building lifetime
**ClosedLoop**	Baseline + diversion of EoL scrap through BOF route + alternative sector split
**ClosedLoop_HighRec**	ClosedLoop + HighRecovery combined
**ClosedLoop_lifetime**	ClosedLoop + 30% lifetime increase
**ClosedLoop_Lt_HighRec**	ClosedLoop + HighRecovery combined + 30% lifetime increase
**NoTrade_LowLoss**	Baseline + no trade + HighRecovery + HighRemeltingYield
**No_EoL_Scrap_Trade**	Baseline + no trade of EoL products and metal scraps
**No_Metal_Product_Trade**	Baseline + no trade of secondary metals and consumer products

Table S20: Scenario storylines.

Scenario name	Scenario storyline
**Baseline**	No change in trade patterns, only already anticipated improvements in process efficiencies
**HighRecovery**	Scenario where strong incentives toward high recovery rates are in place, e.g., by target values for industrial processes set by governments
**HighRecovery_lifetime**	Strong incentives for high recovery AND longer lifetimes, could be the result of a society-wide movement towards material efficiency
**ClosedLoop**	Scenario with push toward high-quality recycling, closed recycling loops on different steel quality levels
**ClosedLoop_HighRec**	Push for low losses AND Closed loop recycling
**ClosedLoop_lifetime**	Push for closed loop recycling AND longer product lifetimes
**ClosedLoop_Lt_HighRec**	All improvement options combined, maximum technical potential for circular material flows
**NoTrade_LowLoss**	Scenario were export barriers are very high and losses are kept low due to material scarcity concerns
**No_EoL_Scrap_Trade**	Scenario were export barriers for steel scrap are very high due to material scarcity concerns or policies for a low carbon steel cycle
**No_Metal_Product_Trade**	Scenario were export barriers are very high for secondary metal and products due to policies that encourage use of recycled material

ADDITIONAL RESULTS

To study the effects of multiple product life cycles and international trade on steel quality and losses in different regions three different baseline scenarios were constructed. In each baseline scenario, a passenger car was registered in the use phase in 2015. In each baseline the location of the registration was in a different regions, and Germany, Japan, and the US were chosen as test cases.

For each of these three baselines the MaTrace Global model was run for all 13 sensitivity cases and for the 10 scenarios.

We present additional results for the consumption flows of steel in selected scenarios, the distribution of stocks across regions and products, the sensitivity analysis, and the scenario analysis.

The functional unit for each scenario is one ton of steel, embedded in final products of different categories.

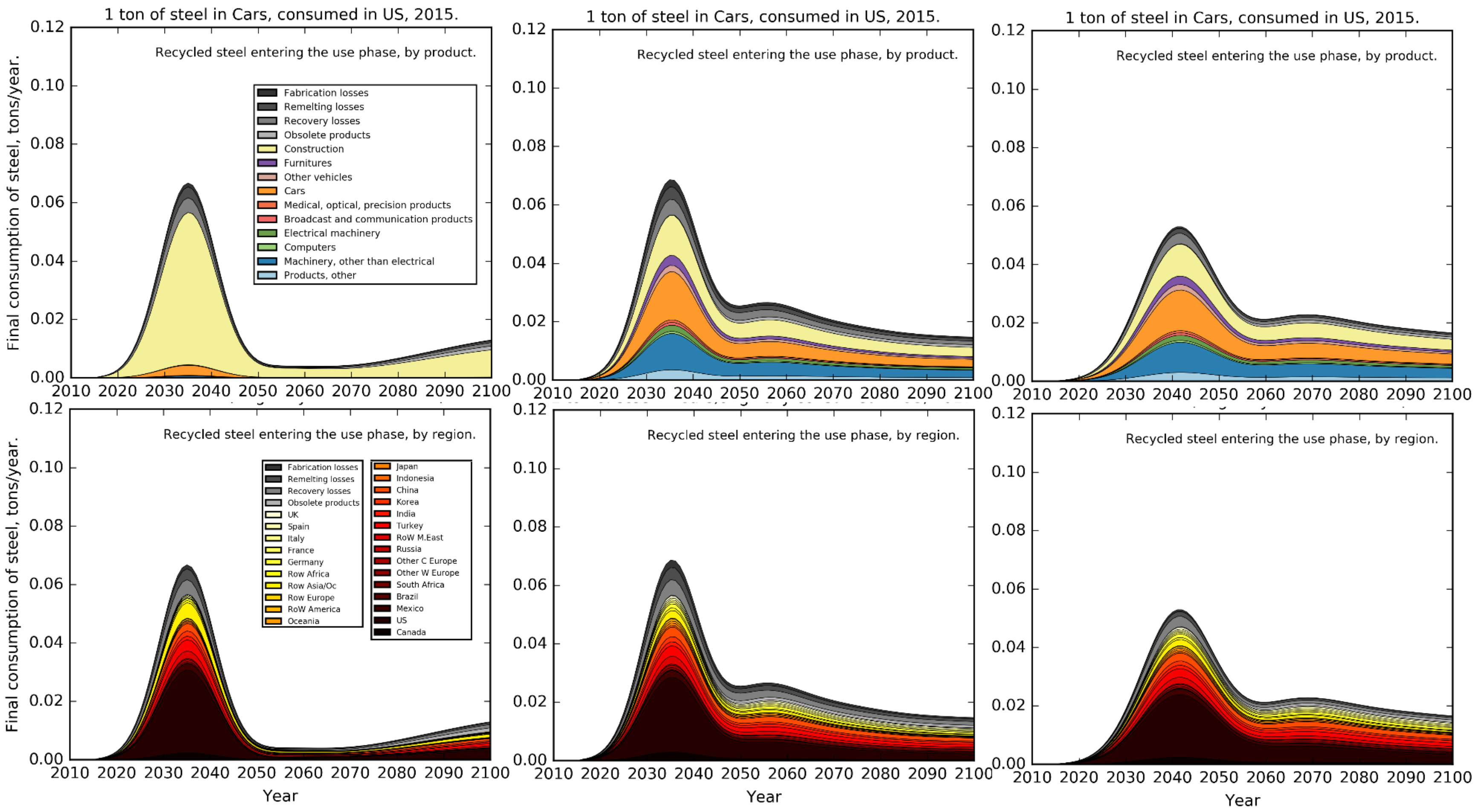

Figure S2: Recycled steel entering the use phase after 1 ton of steel in passenger cars was registered in 2015. Results for registration of the car in the US, scenarios Baseline (left), ClosedLoop (middle), and ClosedLoop_Lt_HighRecovery (right). The upper row shows a breakdown into product groups and loss types, and the lower breakdown shows the regions where the consumption takes place.

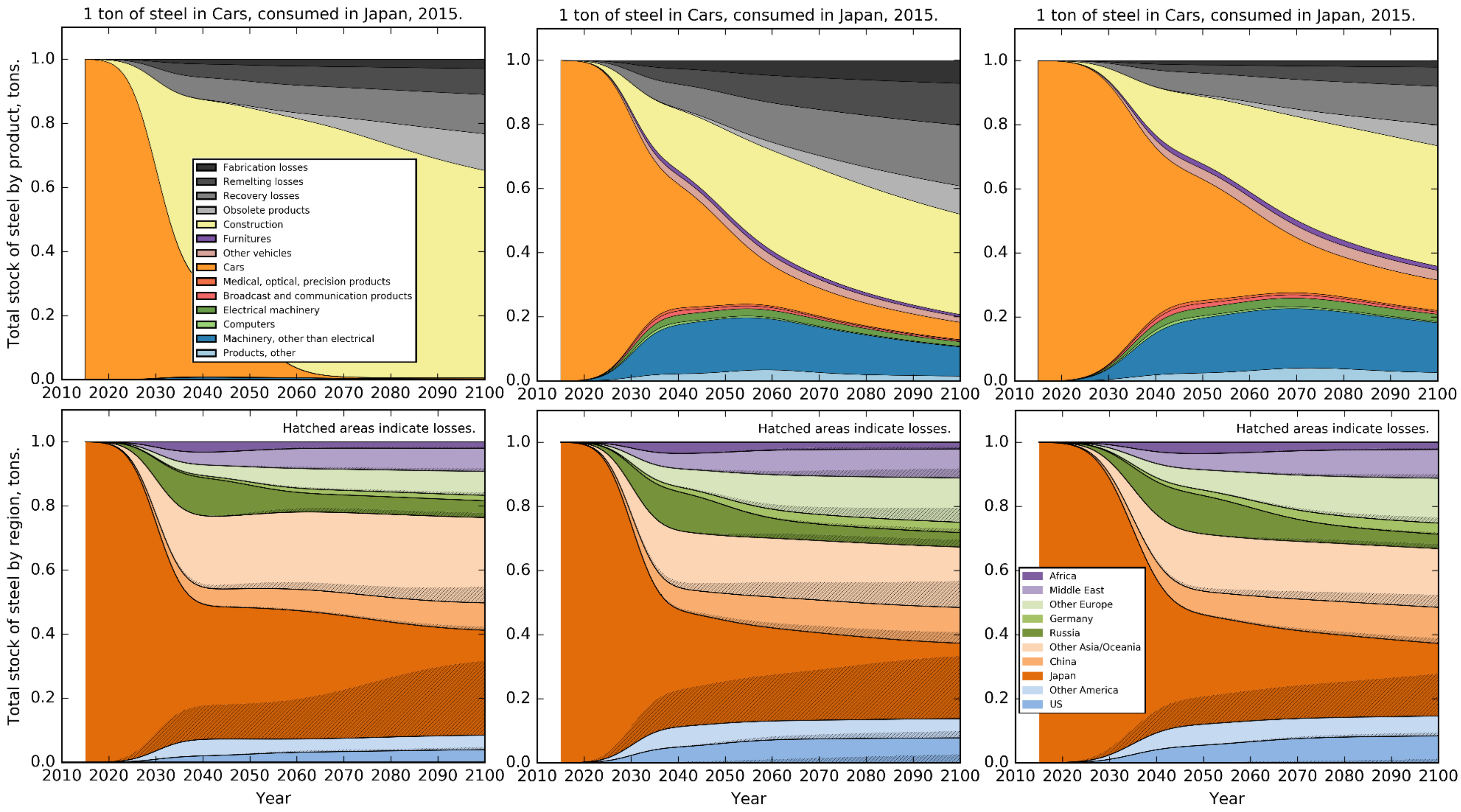

Figure S3: Breakdown of steel stocks after 1 ton of steel in passenger cars was registered in 2015. Results for registration of the car in Japan, scenarios Baseline (left), ClosedLoop (middle), and ClosedLoop_Lt_HighRecovery (right). The upper row shows a breakdown into product groups and loss types, and the lower breakdown shows the regions where the material is located.

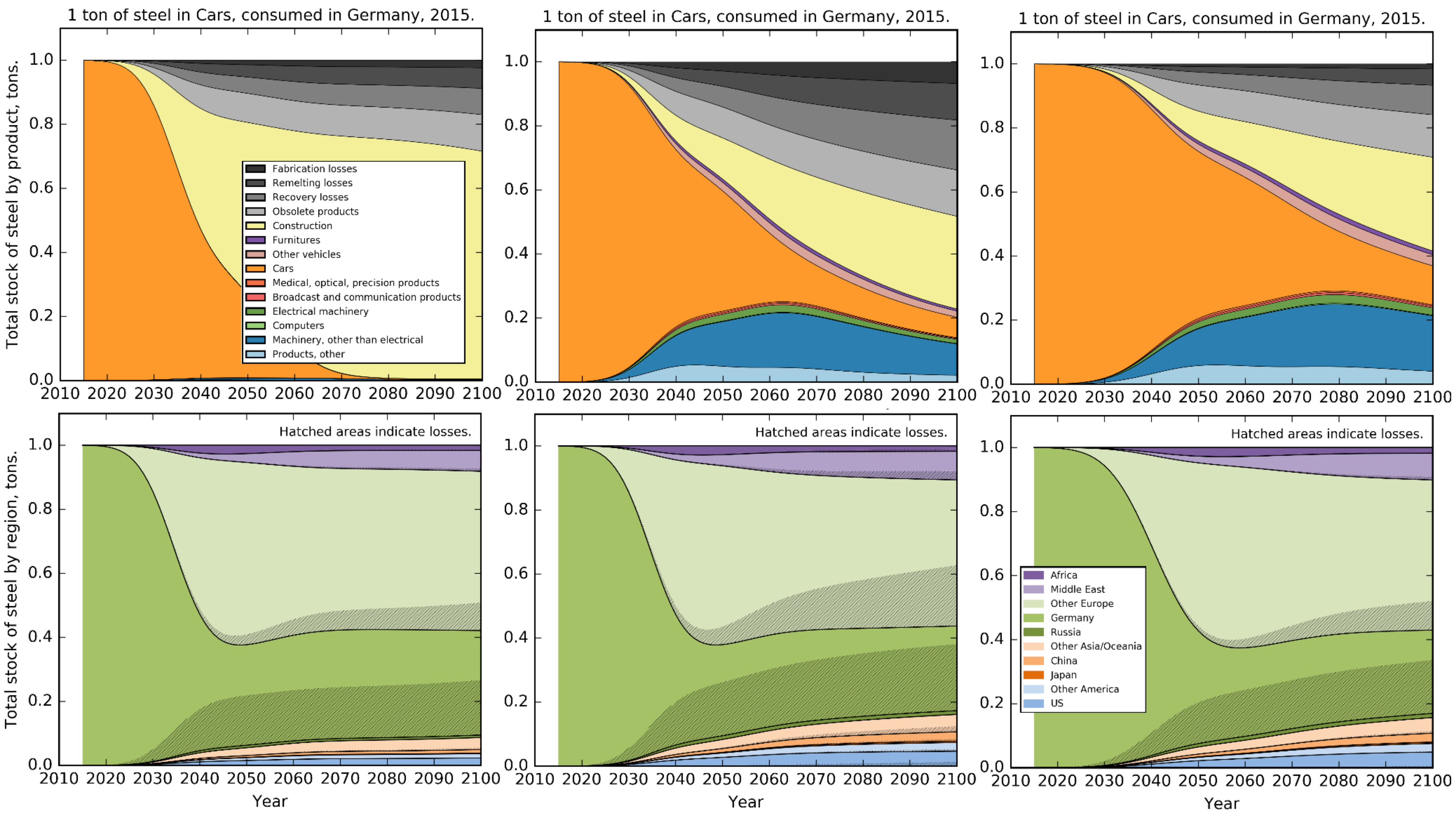

Figure S4: Breakdown of steel stocks after 1 ton of steel in passenger cars was registered in 2015. Results for registration of the car in Germany, scenarios Baseline (left), ClosedLoop (middle), and ClosedLoop_Lt_HighRecovery (right). The upper row shows a breakdown into product groups and loss types, and the lower breakdown shows the regions where the material is located.

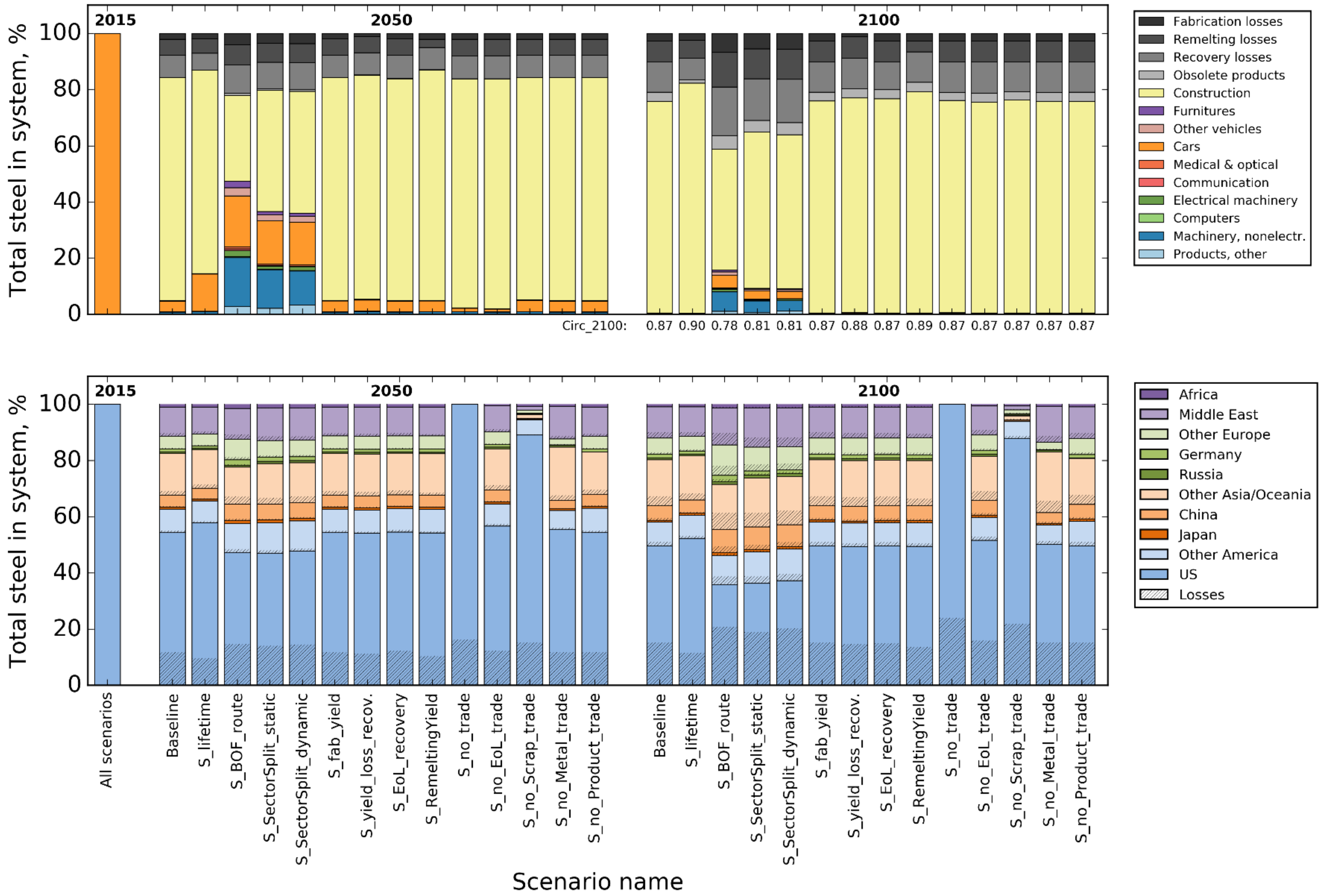

Figure S5: Sensitivity analysis of the product and regional distribution of steel stocks after 1 ton of steel in passenger cars was registered in 2015. Results for registration of the car in the US. The upper row shows a breakdown into product groups and loss types, and the lower breakdown shows the regions where the material is located. Losses are indicated with gray colors in the upper row and with hatchings in the lower row.

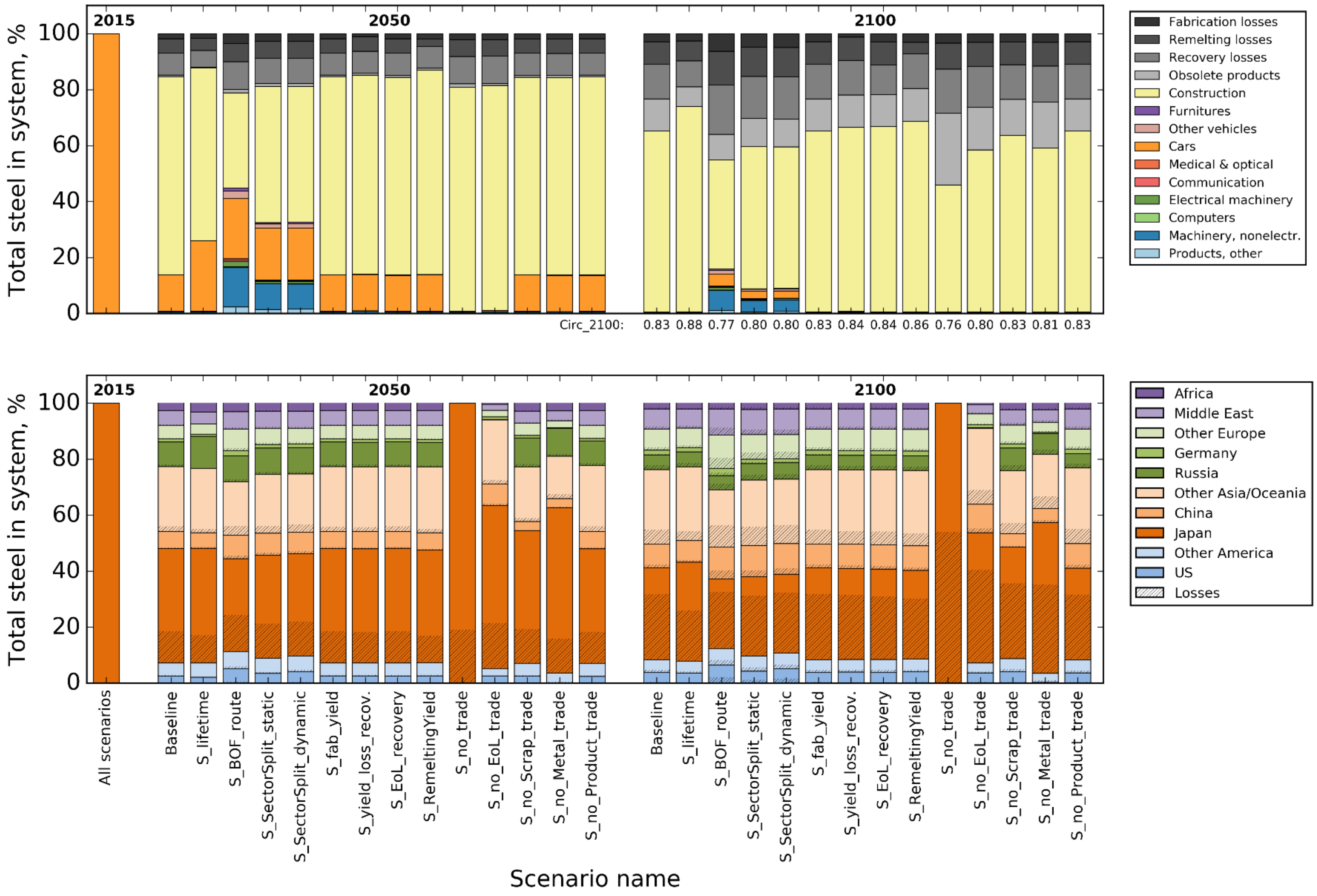

Figure S6: Sensitivity analysis of the product and regional distribution of steel stocks after 1 ton of steel in passenger cars was registered in 2015. Results for registration of the car in Japan. The upper row shows a breakdown into product groups and loss types, and the lower breakdown shows the regions where the material is located. Losses are indicated with gray colors in the upper row and with hatchings in the lower row.

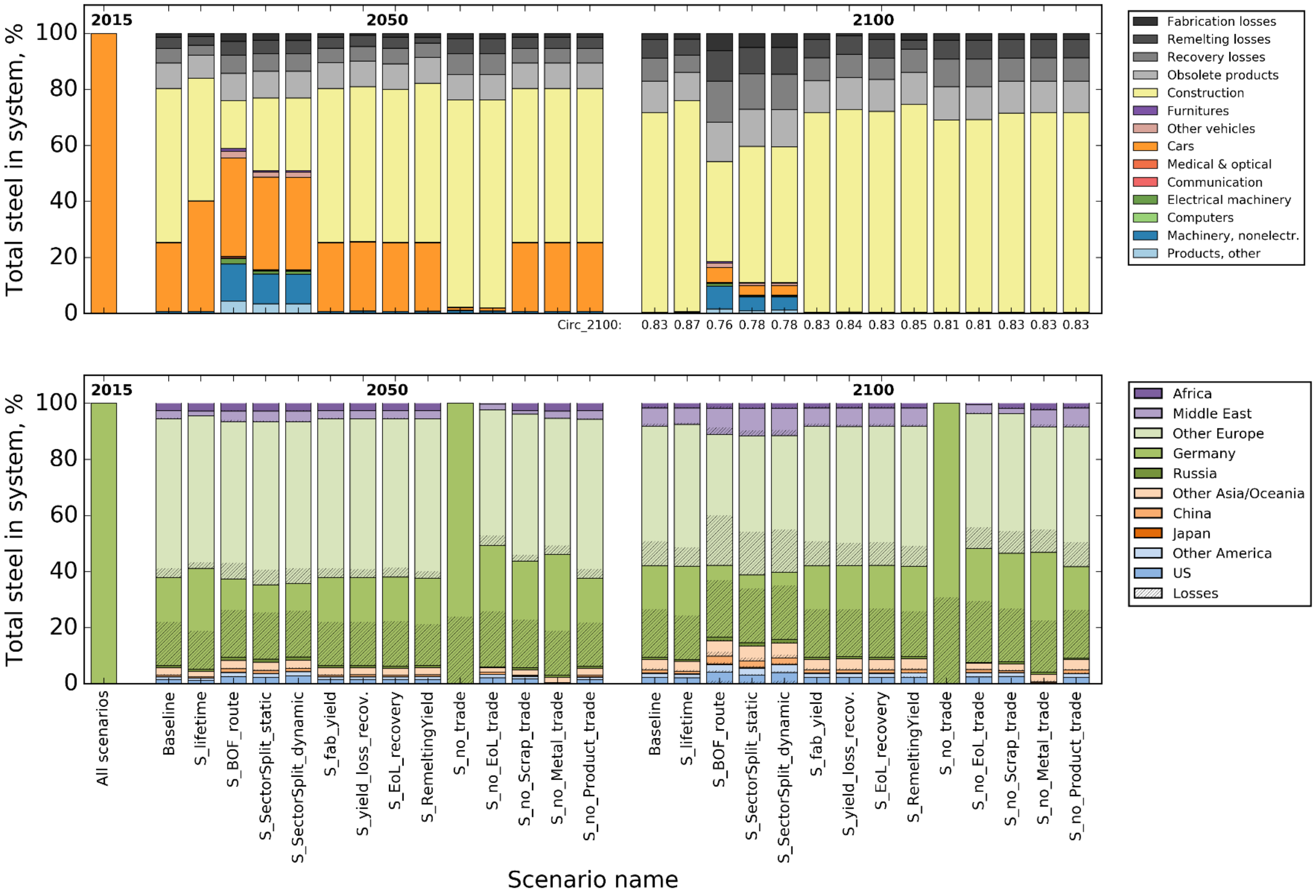

Figure S7: Sensitivity analysis of the product and regional distribution of steel stocks after 1 ton of steel in passenger cars was registered in 2015. Results for registration of the car in Germany. The upper row shows a breakdown into product groups and loss types, and the lower breakdown shows the regions where the material is located. Losses are indicated with gray colors in the upper row and with hatchings in the lower row.

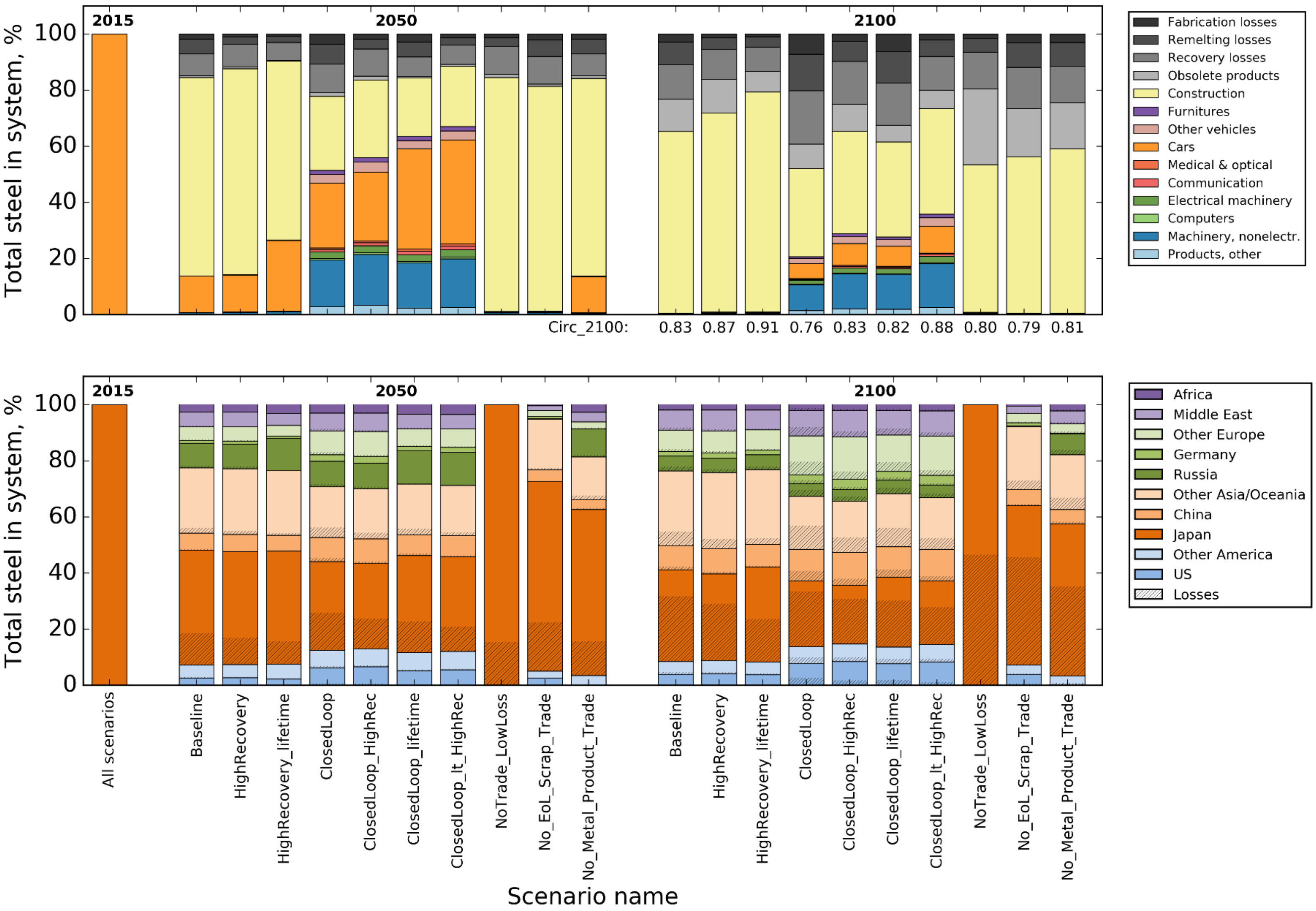

Figure S8: Scenario analysis of the product and regional distribution of steel stocks after 1 ton of steel in passenger cars was registered in 2015. Results for registration of the car in Japan. The upper row shows a breakdown into product groups and loss types, and the lower breakdown shows the regions where the material is located. Losses are indicated with gray colors in the upper row and with hatchings in the lower row.

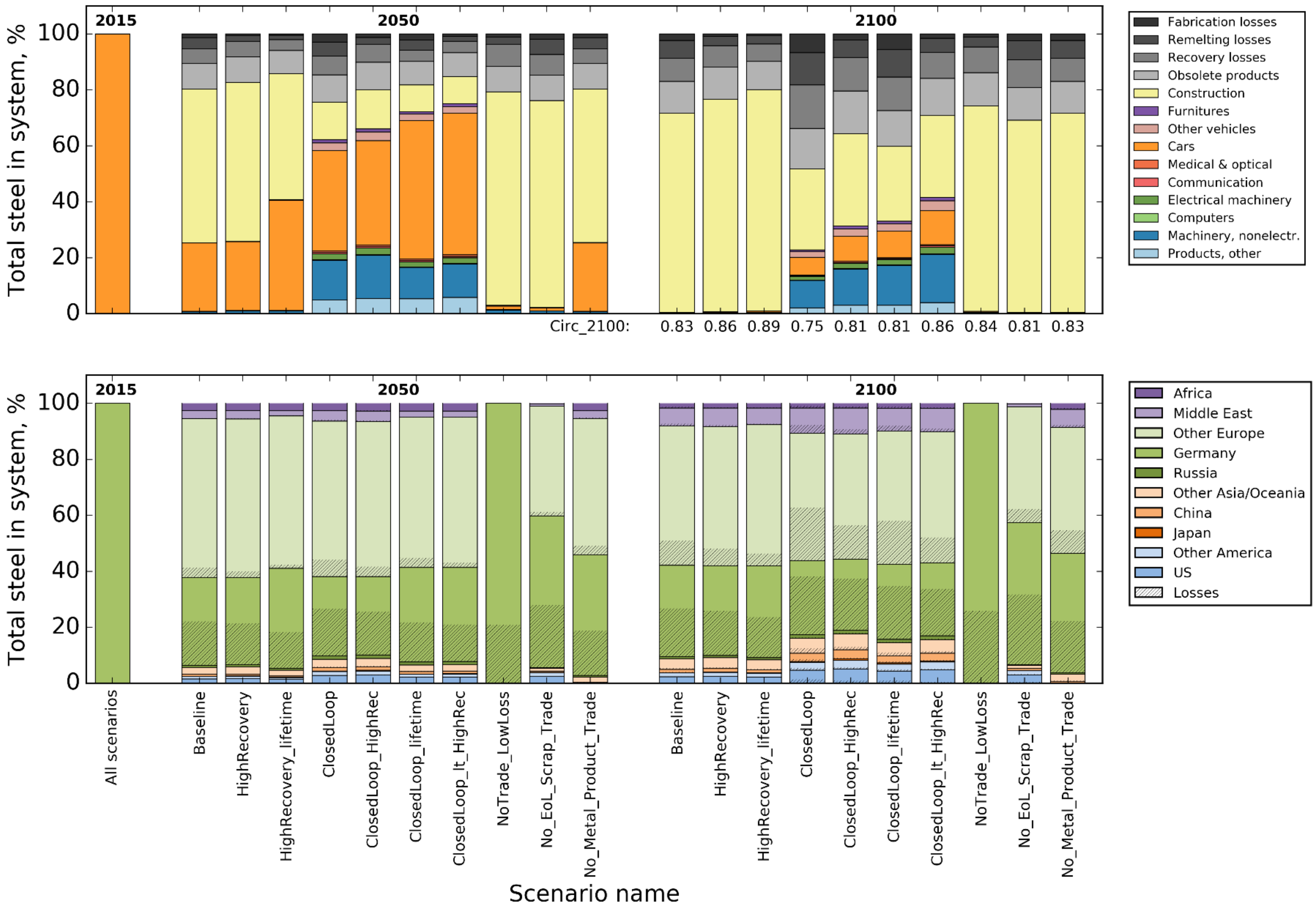

Figure S9: Scenario analysis of the product and regional distribution of steel stocks after 1 ton of steel in passenger cars was registered in 2015. Results for registration of the car in Germany. The upper row shows a breakdown into product groups and loss types, and the lower breakdown shows the regions where the material is located. Losses are indicated with gray colors in the upper row and with hatchings in the lower row.

The situation for a car registered in Germany in 2015 (Figure S9) looks slightly different than for the US (Figure 5 in the paper) and Japan (Figure S8), mainly because more than 50% of all EoL vehicles are exported and re-used in other regions. Already by 2050, about 50% of the steel has moved to other European countries, most of which lie in Eastern Europe, the main importer of EoL vehicles from Germany. In the case of Germany, the contribution of trade of EoL products and scrap to the distribution of the steel across regions is about the same as the contribution of trade of secondary steel and products.

Although many German cars get a second life in other regions, cumulative losses by the end of the century are higher than in the US case, and the Circ(2100) index for Germany is therefore slightly smaller than the index of the US. The main reason for the higher losses in the German case is that data sources report that in some years, up to 50% of German EoL cars have an unknown fate (Kohlmeyer, 2012), which, together with other information, led us to the assumption that 9% of German EoL vehicles end up as obsolete stocks, whereas this fraction is 0% according to US statistics. More complete and reliable EoL product statistics will help to produce more realistic (rather than just plausible) scenarios for MaTrace and other prospective material flow models.

GUIDE TO THE MATRACE GLOBAL MODEL IN PYTHON

This paper comes with a second supplementary file, a zip archive ‘RECYCL-D-16-00862_SI2_20160706.zip’. This archive contains a folder ‘MaTrace Global 20160706’ with three subfolders:

- A folder ‘Data’, where the Excel workbook ‘MaTrace_Global_InData.xlsx’ with the above described parameters is located
- A folder ‘Results’, which is empty and where the scripts store the model results
- A folder ‘Scripts’, which contains the main model script ‘MaTrace_Global_Main.py’ and the scenario intercomparison script ‘MaTrace_Global_ScenarioCompare.py’.

To run the Matrace model, one needs to extract the zip folder and copy its content to a convenient location. Then, in **line 80** of the main script, the path of the MaTrace Global model folder needs to be specified. The MaTrace Model script is a standalone script, which apart from standard Python modules does not need further software.

To run MaTrace Global for a specific parameter constellation, one needs to

- **Define** this constellation in columns H-V of the sheet ‘Parameter_Overview’ of the MaTrace Global datafile. Column H contains the scenario name, column I the scenario description, column J the modus of the model run (at the moment, only ‘TraceSingleProduct’ is supported), column K the start year (e.g., 2015), column J the time horizon (e.g., 2100), column M the test product (at the moment, only ‘Car’ is supported), column N the country index of where the test product is consumed initially (1-25), and columns O-V contain the different improvement options or alternative values for the model parameters that are described above and that can be selected. Row 3 contains comments that describe the possible valid entries for these columns.
- **Select** this constellation by indicating the index number (column G) of the parameter constellation in cell **C4**.
- **Run** the MaTrace Global main script.

The script will then create a subfolder in the results folder with the name structure ScenarioName_DateStamp. In this folder, a copy of the script, a copy of the data file, the figures, the model results as .mat and .xls files, and the log file as .html file are stored.

To compare different MaTrace Global scenarios, the script ‘MaTrace_Global_ScenarioCompare.py’ can be used. Also here, in **line 80**, the path of the MaTrace Global model folder needs to be specified. The variable ScenList (line 182 ff) contains the names of the folders of the scenario runs that are to be compared, and the scrip will read the corresponding results from the Result folder. The variable ScenList_Names_Plot contains short names of these scenarios for display in the figures. Each of the figures created in the script is controlled by one or more ‘ScenSel’ parameters, which contain the indices of the scenario runs in ScenList that are to be used for making the comparison plots.

Note: The MaTrace Global scripts can be run and modified for research and teaching purposes. No additional support is provided by the authors. Check https://github.com/IndEcol/Dashboard for updates on the MaTrace Global model code.

REFERENCES

Allwood, J. M., Cullen, J. M., Carruth, M. A., Cooper, D. R., McBrien, M., Milford, R. L., … Patel, A. C. H. (2012). *Sustainable Materials: with both eyes open*. Cambridge, UK: UIT Cambridge, UK.

Cullen, J. M., Allwood, J. M., & Bambach, M. D. (2012). Mapping the global flow of steel: from steel making to end-use goods. *Environmental Science & Technology*, *46*(24), 13048–55. doi:10.1021/es302433p

Ellen MacArthur Foundation. (2015). *Circularity Indicators - An Approach to Measuring Circularity*. Cowes, UK.

Gaulier, G., & Zignago, S. (2010). BACI : International Trade Database at the Product-level The 1994-2007 Version.

Graedel, T. E., Allwood, J. M., Birat, J.-P., Buchert, M., Hagelüken, C., Reck, B. K., … Sonnemann, G. (2011). What Do We Know About Metal Recycling Rates? *Journal of Industrial Ecology*, *15*(3), 355–366. doi:10.1111/j.1530-9290.2011.00342.x

Graedel, T. E., Barr, R., Chandler, C., Chase, T., Choi, J., Christoffersen, L., … Zhu, C. (2012). Methodology of metal criticality determination. *Environmental Science & Technology*, *46*(2), 1063–70. doi:10.1021/es203534z

Graedel, T. E., Harper, E. M., Nassar, N. T., Nuss, P., & Reck, B. K. (2015). Criticality of metals and metalloids. *Proceedings of the National Academy of Sciences of the United States of America*, *112*(14), 4257–62. doi:10.1073/pnas.1500415112

Ignatenko, O., van Schaik, A., & Reuter, M. A. (2008). Recycling system flexibility: the fundamental solution to achieve high energy and material recovery quotas. *Journal of Cleaner Production*, *16*(4), 432–449. doi:10.1016/j.jclepro.2006.07.048

Kagawa, S., Nakamura, S., Kondo, Y., Matsubae, K., & Nagasaka, T. (2015). Forecasting Replacement Demand of Durable Goods and the Induced Secondary Material Flows. *Journal of Industrial Ecology*, *19*(1), 10–19. doi:10.1111/jiec.12184

Kohlmeyer, R. (2012). Aktuelles zur Verwendung von Altfahrzeugen (Updates on the fate of end-of-life vehicles). Retrieved April 3, 2016, from https://www.umweltbundesamt.de/sites/default/files/medien/421/dokumente/abfall-ressourcen_produktverantwortung_altfahrzeuge_vortrag-ihk.pdf

Milford, R. L., Pauliuk, S., Allwood, J. M., & Müller, D. B. (2013). The Roles of Energy and Material Efficiency in Meeting Steel Industry CO2 Targets. *Environmental Science & Technology*, *47*(7), 3455–3462. doi:10.1021/es3031424

Miller, R. E., & Blair, P. D. (2009). *Input-Output Analysis Foundations and Extensions*. New York: Cambridge University Press.

Müller, D. B., Cao, J., Kongar, E., M., A., Weiner, P.-H., & Graedel, T. E. (2007). *Service Lifetimes of Mineral End Uses*. (U. S. G. Survey, Ed.)*Minerals Resources External Research Program*. New Haven: Yale University.

Murakami, S., Oguchi, M., Tasaki, T., Daigo, I., & Hashimoto, S. (2010). Lifespan of Commodities , Part I - The Creation of a Database and Its Review. *Journal of Industrial Ecology*, *14*(4), 598–612. doi:10.1111/j.1530-9290.2010.00250.x

Nakamura, S., Kondo, Y., Kagawa, S., Matsubae, K., Nakajima, K., & Nagasaka, T. (2014). MaTrace: Tracing the Fate of Materials over Time and Across Products in Open-Loop Recycling. *Environmental Science & Technology*. doi:10.1021/es500820h

Nakamura, S., Nakajima, K., Kondo, Y., & Nagasaka, T. (2007). The Waste Input-Output Approach to Materials Flow Analysis Concepts and Application to Base Metals. *Journal of Industrial Ecology*, *11*(4), 50–63. doi:10.1162/jiec.2007.1290

Ohno, H., Matsubae, K., Nakajima, K., Kondo, Y., Nakamura, S., & Nagasaka, T. (2015). Toward the efficient recycling of alloying elements from end of life vehicle steel scrap. *Resources, Conservation and Recycling*, *100*, 11–20. doi:10.1016/j.resconrec.2015.04.001

Ohno, H., Matsubae, K., Nakajima, K., Nakamura, S., & Nagasaka, T. (2014). Unintentional Flow of Alloying Elements in Steel during Recycling of End-of-Life Vehicles. *Journal of Industrial Ecology*, *18*(2), 242–253. doi:10.1111/jiec.12095

Pauliuk, S., Dhaniati, N. M. a, & Müller, D. B. (2012). Reconciling sectoral abatement strategies with global climate targets: the case of the Chinese passenger vehicle fleet. *Environmental Science & Technology*, *46*(1), 140–7. doi:10.1021/es201799k

Pauliuk, S., Milford, R. L., Müller, D. B., & Allwood, J. M. (2013). The Steel Scrap Age. *Environmental Science & Technology*, *47*(7), 3448–3454. doi:10.1021/es303149z

Pauliuk, S., Wang, T., & Müller, D. B. (2012). Moving toward the circular economy: the role of stocks in the Chinese steel cycle. *Environmental Science & Technology*, *46*(1), 148–54. doi:10.1021/es201904c

Reuter, M. A., van Schaik, A., Ignatenko, O., & de Haan, G. J. (2006). Fundamental limits for the recycling of end-of-life vehicles. *Minerals Engineering*, *19*(5), 433–449. doi:10.1016/j.mineng.2005.08.014

Smith, N. J., & McDonald, G. W. (2011). Estimation of Symmetric Input–Output Tables: an Extension To Bohlin and Widell. *Economic Systems Research*, *23*(1), 49–72. doi:10.1080/09535314.2010.534977

Stubbles, J. (n.d.). THE BASIC OXYGEN STEELMAKING (BOS) PROCESS. Retrieved April 9, 2016, from http://www.steel.org/making-steel/how-its-made/processes/processes-info/the-basic-oxygen-steelmaking-process.aspx

Suh, S., Weidema, B. P., Schmidt, J. H., & Heijungs, R. (2010). Generalized Make and Use Framework for Allocation in Life Cycle Assessment. *Journal of Industrial Ecology*, *14*(2), 335–353. doi:10.1111/j.1530-9290.2010.00235.x

van der Voet, E., Kleijn, R., Huele, R., Ishikawa, M., & Verkuijlen, E. (2002). Predicting future emissions based on characteristics of stocks. *Ecological Economics*, *41*(2), 223–234.

Wood, R., Stadler, K., Bulavskaya, T., Lutter, S., Giljum, S., de Koning, A., … Tukker, A. (2014). Global Sustainability Accounting—Developing EXIOBASE for Multi-Regional Footprint Analysis. *Sustainability*, *7*(1), 138–163. doi:10.3390/su7010138

World Steel Association. (2009). *Yield improvement in the steel industry*. Brussels/Beijing: WorldSteel Association.

World Steel Association. (2010). *The three Rs of sustainable steel*. Brussels/Beijing: WorldSteel Association.
