# Supplementary material for: Regional distribution and losses of end-of-life steel throughout multiple product life cycles—Insights from the global multiregional MaTrace model
Source: Resour Conserv Recycl. 2017 Jan;116:84–93. doi: 10.1016/j.resconrec.2016.09.029 (PMC5302007; doi:10.1016/j.resconrec.2016.09.029)
Supplement: Supplementary file 2 [file mmc2.zip › MaTrace Global 20160706/MaTrace_Global_SI2_Guide.docx]

*Resources Conservation and Recycling*

Regional distribution and losses of end-of-life steel throughout multiple product life cycles — Insights from the global multiregional MaTrace model

Stefan Pauliuk,^1,*^ Yasushi Kondo,^2^ Shinichiro Nakamura, ^2^ and Kenichi Nakajima^3^

^1^ Faculty of Environment and Natural Resources, University of Freiburg, Germany

^2^ Graduate School of Economics, Waseda University, Tokyo, Japan

^3^ Center for Material Cycles and Waste Management Research, National Institute for Environmental Studies, Tsukuba, Japan

***Guide to Supplementary Material 2 (of 2)***

To whom correspondence should be addressed:

*) Stefan Pauliuk

Faculty of Environment and Natural Resources, University of Freiburg, D-79106 Freiburg, Germany

[stefan.pauliuk@indecol.uni-freiburg.de](mailto:stefan.pauliuk@indecol.uni-freiburg.de); phone+49-761-203-98726; fax +49-761-203-3600

# GUIDE TO THE MATRACE GLOBAL MODEL IN PYTHON

This paper comes with a second supplementary file, a zip archive ‘RECYCL-D-16-00862_SI2_20160706.zip’. This archive contains a folder ‘MaTrace Global 20160706’ with three subfolders:

- A folder ‘Data’, where the Excel workbook ‘MaTrace_Global_InData.xlsx’ with the above described parameters is located
- A folder ‘Results’, which is empty and where the scripts store the model results
- A folder ‘Scripts’, which contains the main model script ‘MaTrace_Global_Main.py’ and the scenario intercomparison script ‘MaTrace_Global_ScenarioCompare.py’.

To run the Matrace model, one needs to extract the zip folder and copy its content to a convenient location. Then, in **line 80** of the main script, the path of the MaTrace Global model folder needs to be specified. The MaTrace Model script is a standalone script, which apart from standard Python modules does not need further software.

To run MaTrace Global for a specific parameter constellation, one needs to

- **Define** this constellation in columns H-V of the sheet ‘Parameter_Overview’ of the MaTrace Global datafile. Column H contains the scenario name, column I the scenario description, column J the modus of the model run (at the moment, only ‘TraceSingleProduct’ is supported), column K the start year (e.g., 2015), column J the time horizon (e.g., 2100), column M the test product (at the moment, only ‘Car’ is supported), column N the country index of where the test product is consumed initially (1-25), and columns O-V contain the different improvement options or alternative values for the model parameters that are described above and that can be selected. Row 3 contains comments that describe the possible valid entries for these columns.
- **Select** this constellation by indicating the index number (column G) of the parameter constellation in cell **C4**.
- **Run** the MaTrace Global main script.

The script will then create a subfolder in the results folder with the name structure ScenarioName_DateStamp. In this folder, a copy of the script, a copy of the data file, the figures, the model results as .mat and .xls files, and the log file as .html file are stored.

To compare different MaTrace Global scenarios, the script ‘MaTrace_Global_ScenarioCompare.py’ can be used. Also here, in **line 80**, the path of the MaTrace Global model folder needs to be specified. The variable ScenList (line 182 ff) contains the names of the folders of the scenario runs that are to be compared, and the scrip will read the corresponding results from the Result folder. The variable ScenList_Names_Plot contains short names of these scenarios for display in the figures. Each of the figures created in the script is controlled by one or more ‘ScenSel’ parameters, which contain the indices of the scenario runs in ScenList that are to be used for making the comparison plots.

**Note:** The MaTrace Global scripts can be run and modified for research and teaching purposes. No additional support is provided by the authors. Check <https://github.com/IndEcol/Dashboard> for updates on the MaTrace Global model code.
